# SQSTM1/p62 UFMylation Enhances Autophagic Clearance of Pathogenic Mutant Huntingtin

**DOI:** 10.7150/ijbs.127110

**Published:** 2026-05-18

**Authors:** Xiaohui Wang, Xiaowei Lv, Honglv Jiang, Wenyun Zhu, Lindong Cao, Liang Zhou, Fang Lin, Rong Deng, Li-Fang Hu, Jingjing Ma, Jia-Bin Li, Guoqiang Xu

**Affiliations:** 1 Jiangsu Key Laboratory of Drug Discovery and Translational Research for Brain Diseases and College of Pharmaceutical Sciences, The Fourth Affiliated Hospital of Soochow University, Jiangsu Province Engineering Research Center of Precision Diagnostics and Therapeutics Development, Jiangsu Key Laboratory of Preventive and Translational Medicine for Major Chronic Non-communicable Diseases, Suzhou Key Laboratory of Drug Research for Prevention and Treatment of Hyperlipidemic Diseases, Soochow University, Suzhou, Jiangsu 215123, China.; 2 Department of Neurology and Clinical Research Center of Neurological Disease, The Second Affiliated Hospital of Soochow University, Suzhou, Jiangsu 215004, China.; 3 Jiangsu Key Laboratory of Drug Discovery and Translational Research for Brain Diseases, Institute of Neuroscience, Soochow University, Suzhou, Jiangsu 215123, China.; 4 Department of Pharmacy, The Fourth Affiliated Hospital of Soochow University, Suzhou Dushu Lake Hospital, Medical Center of Soochow University, Suzhou, Jiangsu 215123, China.; 5 State Key Laboratory of Neurology and Oncology Drug Development, Jiangsu, China.; 6 Biomedical Basic Research Center (BBRC) of Jiangsu Province, Medical College of Soochow University, Suzhou, Jiangsu 215123, China.; 7 Suzhou International Joint Laboratory for Diagnosis and Treatment of Brain Diseases, College of Pharmaceutical Sciences, Soochow University, Suzhou, Jiangsu 215123, China.; 8 MOE Key Laboratory of Geriatric Diseases and Immunology, Suzhou Medical College of Soochow University, Suzhou, Jiangsu 215123, China.; 9 Suzhou Key Laboratory of Geriatric Neurological Disorders, the First People's Hospital of Taicang, Taicang Affiliated Hospital of Soochow University, Suzhou, Jiangsu, 215400, China.

**Keywords:** SQSTM1/p62, UFMylation, quantitative proteomics, autophagic degradation, Huntington's disease, mutant huntingtin

## Abstract

Ubiquitin-fold modifier 1 (UFM1) covalently modifies protein substrates (UFMylation) and alters their biological functions. Genetic screening disclosed that enzymes in the UFMylation system play critical roles in regulating autophagy. However, it is still elusive which protein is UFMylated and how this modification modulates autophagy. Here, our quantitative proteomics and biochemical experiments identify SQSTM1/p62 as a UFMylation substrate and discover its two major UFMylation sites, K420 and K435. Mutating them to Arg (p62^2KR^) completely abolishes the effect of p62 on autophagic activity. Fusion of UFM1^ΔC4^ to p62^2KR^ (p62^2KR^-UFM1^ΔC4^) restores the p62-mediated pathogenic autophagic degradation in primary cortical neurons and Huntington's disease mouse striatum. Mechanistically, p62 UFMylation enhances its interaction with LC3, augments autophagic flux, and eliminates pathogenic mutant huntingtin. Collectively, this work discovers a new post-translational modification, UFMylation, on p62 and establishes this modification as a key regulator of autophagy that promotes the clearance of mutant huntingtin, offering a potential target for therapeutic intervention.

## Introduction

Autophagy-lysosome pathway (ALP) is one of the cellular protein degradation modalities in eukaryotes, which sustains cellular homeostasis by degrading unfolded, misfolded, or unwanted proteins 1. Typically, the ALP acts primarily to remove protein aggregates, damaged organelles, and intracellular pathogens through the formation of autophagosomes that are subsequently fused with lysosomes for proteolysis. Dysfunction of the ALP is associated with many diseases, including neurodegenerative diseases, cancer, microbial infection, and immune disorders 2-5. Although it has been discovered that autophagy is modulated both at the transcriptional and post-translational levels, the regulatory mechanism of the ALP is not completely understood.

The pathogenic hallmark of Huntington's disease (HD) is the aberrant expansion of the CAG trinucleotide repeats in the huntingtin (HTT) gene. This neurodegenerative disorder is manifested as progressive degeneration in the striatum and cortex, marked by the gradual loss of medium spiny neurons (MSNs) alongside pyramidal neurons, ultimately impairing cortico-striatal circuitry 6. Emerging studies on the pathogenesis of HD have demonstrated that autophagosome-tethering compounds effectively facilitate the clearance of mutant HTT (mHTT) by selectively targeting protein aggregates to autophagic machinery 7. This observation suggests that augmenting selective autophagy flux to enhance the clearance of mHTT constitutes a promising HD intervention paradigm, presenting both theoretical importance and translational potential for developing new therapeutics for neurodegenerative diseases.

SQSTM1/p62 (sequestosome 1, hereafter p62) is a critical autophagy receptor that recruits protein aggregates to autophagosomes through its N-terminal Phox1 and Bem1p domain, LC3-interacting region (LIR), and ubiquitin-associated domain (UBA), thereby promoting the lysosomal degradation of intracellular protein aggregates 8-11. It is well established that p62 acts as a receptor to colocalize with mHTT and mediates its autophagic degradation 12-15. The function of p62 is precisely controlled by a diverse array of post-translational modifications (PTMs), such as phosphorylation 16, 17, acetylation 18, ubiquitination 19-21, and FAT10ylation 22. These modifications modulate the autophagic clearance of cargo proteins by orchestrating their affinity to p62, the capacity of target autophagosomes, and the interaction between p62 and LC3.

UFM1 is a ubiquitin-like modifier that has a strikingly conserved tertiary structure resembling ubiquitin 23. UFM1 can also covalently modify protein substrates (UFMylation) through an enzymatic cascade comprising the UFM1-activating enzyme UBA5, the UFM1-conjugating enzyme UFC1, and the UFM1-protein ligase UFL1 24, 25. Interestingly, the DDRGK domain-containing protein 1 (DDRGK1 or UFBP1) is also an important adaptor that promotes protein UFMylation 26. In addition, conjugated UFM1 could be removed by UFSP1 and UFSP2, two UFM1-specific proteases 27-29. Besides the components in the UFMylation system, more than two dozen proteins were discovered to be UFMylated 24. Recent studies have revealed that UFMylation plays diverse roles in many cellular processes, including endoplasmic reticulum (ER) homeostasis 30, protein biogenesis 31, cell cycle progression 32, DNA damage response 33, 34, brain development 35, and cancer-associated signaling pathways 26, 36-39. Emerging evidence indicates that UFMylation modulates protein degradation through the ALP, highlighting its potential as a therapeutic target. Specifically, VCP/p97 UFMylation at K107 enhances the stabilization of beclin-1 and facilitates the formation of the phosphatidylinositol 3-kinase (PI3K)/PtdIns3K complex. This finding indicates that VCP/p97 UFMylation plays a vital regulatory function in autophagy initiation 40. In addition, UFL1 interacts with NLRP3 and mediates its UFMylation during activation, while stabilizing the NLRP3 protein by inhibiting K63-linked polyubiquitination and diminishing its subsequent autophagic degradation 41. However, all of these studies explored the autophagic degradation of UFMylated substrates without delving into the relationship between UFMylation and autophagy.

Previous work revealed that UFL1-deficiency in bone marrow cells attenuated autophagic degradation 42. Autophagy-related protein 8-family proteins (including LC3 in humans) interact with the non-canonical LIR motif in UBA5, regulating its subcellular distribution and biological functions 43. Depletion of UFBP1 hampers the autophagosome-lysosome fusion, which results in the accumulation of autophagosomes and enhanced apoptosis in mouse embryonic fibroblasts 44. CRISPR/Cas9-based genetic screening disclosed that the p62 function was regulated by UFM1 and the components in the UFMylation system 45. Nonetheless, it is elusive whether p62 is UFMylated, and the precise biochemical mechanisms by which UFMylation regulates autophagy have yet to be elucidated.

Here, we employ tandem affinity purification coupled with quantitative proteomics to identify the potential UFMylated proteins and confirm that p62 is mainly UFMylated at K420 and K435. We further demonstrate that the wild-type (WT) p62 but not the UFMylation-deficient mutant promotes the LC3II accumulation in autophagic degradation upon *p62* knockdown in cell lines and in primary cortical neurons. Fusion of a C-terminal truncated UFM1 to the UFMylation-deficient p62 mutant almost completely restores the function of p62 as an autophagy receptor. Mechanistically, p62 UFMylation enhances the binding of p62 to LC3II and thereby promotes autophagic degradation of pathogenic mHTT. Moreover, p62 UFMylation is attenuated in the cortex of transgenic HD mice, and mimicking p62 UFMylation promotes the clearance of mHTT and reinstates the density of MSNs in the striatum of HD mice. Thus, we discover a new PTM, UFMylation, on p62 and reveal a novel function of p62 UFMylation in elevating autophagic activity, offering new insights into developing potential therapeutic strategies for autophagic degradation of pathogenic mHTT by targeting the UFMylation system.

## Results

### Depletion of UFL1 or UBA5 impairs the autophagic flux

**Figure 1:**
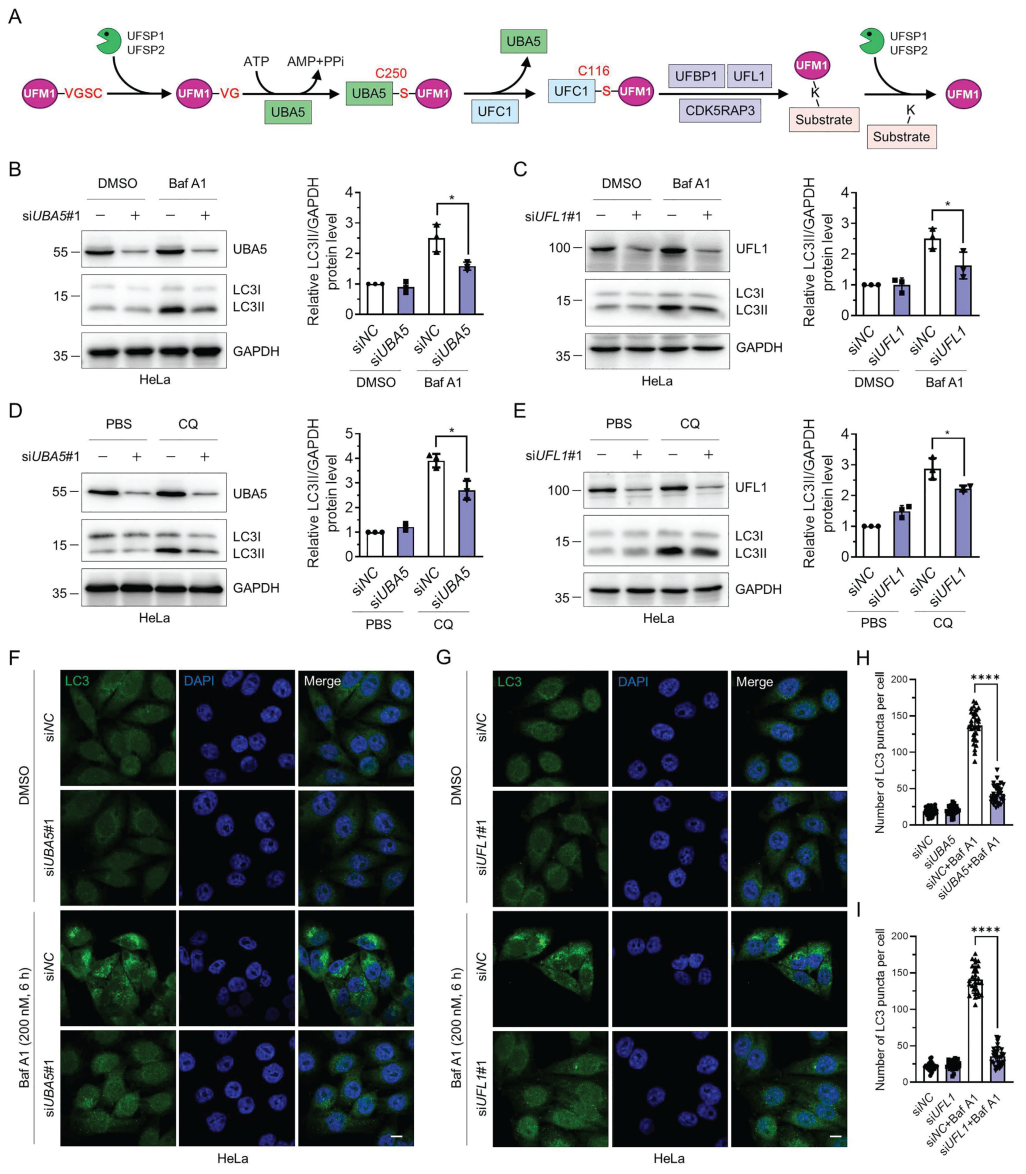
** Knockdown of *UBA5* or* UFL1* attenuates the autophagic activity.** (**A**) The schematic illustration of the UFMylation pathway. UFM1 is synthesized as a precursor which is hydrolyzed by UFSP1/2 at its C-terminus to expose glycine. The mature UFM1 is then activated and subjected to a nucleophilic attack by the Cys250 of UBA5. Then activated UFM1 is transferred to UFC1 Cys116 through a thioester bond. In this process, UFL1 forms a tripartite complex with UFBP1 or CDK5RAP3 and transfers UFM1 to lysine residues of target proteins. UFSP1/2 mediate the deUFMylation by removing UFM1 from substrates. (**B-E**) Knockdown of *UBA5* or *UFL1* reduces autophagic activity. HeLa cells were transfected with si*NC*, si*UBA5*#1, or si*UFL1*#1 for 42 h and treated with bafilomycin A1 (Baf A1, 200 nM for 6 h) (B-C) or chloroquine (CQ, 50 μM for 12 h) (D-E). Cell lysates were immunoblotted for LC3, UBA5, and UFL1. The relative protein levels of LC3II were normalized to GAPDH and quantified. Data were presented as mean ± SD (n = 3 biological replicates), two-way ANOVA with Tukey's multiple comparisons post hoc test, *: *P* < 0.05. (**F**-**I**) LC3 puncta are reduced in *UBA5*- or *UFL1*-depleted HeLa cells. Immunofluorescence staining (F-G) and quantification (H-I) of LC3 in HeLa cells treated as in (B-C). Scale bar, 10 μm. Mean ± SD (n = 3 biological replicates with 30 cells, each point represented the number of puncta in one cell), two-way ANOVA with Tukey's multiple comparisons post hoc test, ****: *P* < 0.0001.

The UFMylation cascade is initiated by cleaving the last two amino acids in UFM1 precursor by UFSP1 and UFSP2. Subsequently, mature UFM1 is covalently linked to target proteins through the UBA5-UFC1-UFL1 tripartite enzymatic reactions. UFBP1 and/or CDK5RAP3 are essential regulators for substrate UFMylation. Furthermore, UFSP1 and UFSP2 regulate the deUFMylation of substrates, rendering UFMylation a reversible modification (Fig. 1A). Previously, it has been reported that UFL1 deficiency impairs autophagic degradation 42. Atg8-family proteins interact with UBA5 and regulate its localization and biological functions 43. However, the regulatory roles of UFL1 or UBA5 in autophagy and the underlying molecular mechanisms are still mysterious. Here, we sought to explore the function of UBA5 and UFL1 in autophagic degradation. Knockdown of either *UBA5* or *UFL1* significantly reduced the LC3II accumulation induced by two late-stage autophagy inhibitors, bafilomycin A1 (Baf A1) and chloroquine (CQ) 46 (Fig. 1B-E), indicating that UBA5 or UFL1 depletion attenuates the autophagic flux. Similar phenomena were observed in MCF-7 cells (Fig. S1A-D). Knockdown of *UBA5* or *UFL1* with another set of siRNAs (si*UBA5*#2 and si*UFL1*#2) also exhibited a similar trend (Fig. S1E-H). Consistently, beclin-1 was also reduced when *UBA5* or *UFL1* was depleted in HeLa cells (Fig. S1I-J). To further explore the role of these two proteins in the regulation of autophagy, we examined LC3 puncta by immunofluorescence in the control (si*NC*), *UBA5*- or *UFL1*-depleted (si*UBA5* or si*UFL1*) HeLa cells. In the control group, the number of LC3 puncta was increased markedly upon the inhibition of autophagic degradation by Baf A1. However, interestingly, Baf A1-induced increase of LC3 puncta was pronouncedly attenuated in the *UBA5*- or *UFL1*-depleted cells, indicating that UBA5 and UFL1 were indeed two general regulators of autophagy (Fig. 1F-I and Fig. S1K-L). Thus, these findings indicate that depletion of UBA5 or UFL1 inhibits autophagic flux.

### p62 is a bona fide UFMylation substrate and is mainly UFMylated on K420 and K435

**Figure 2:**
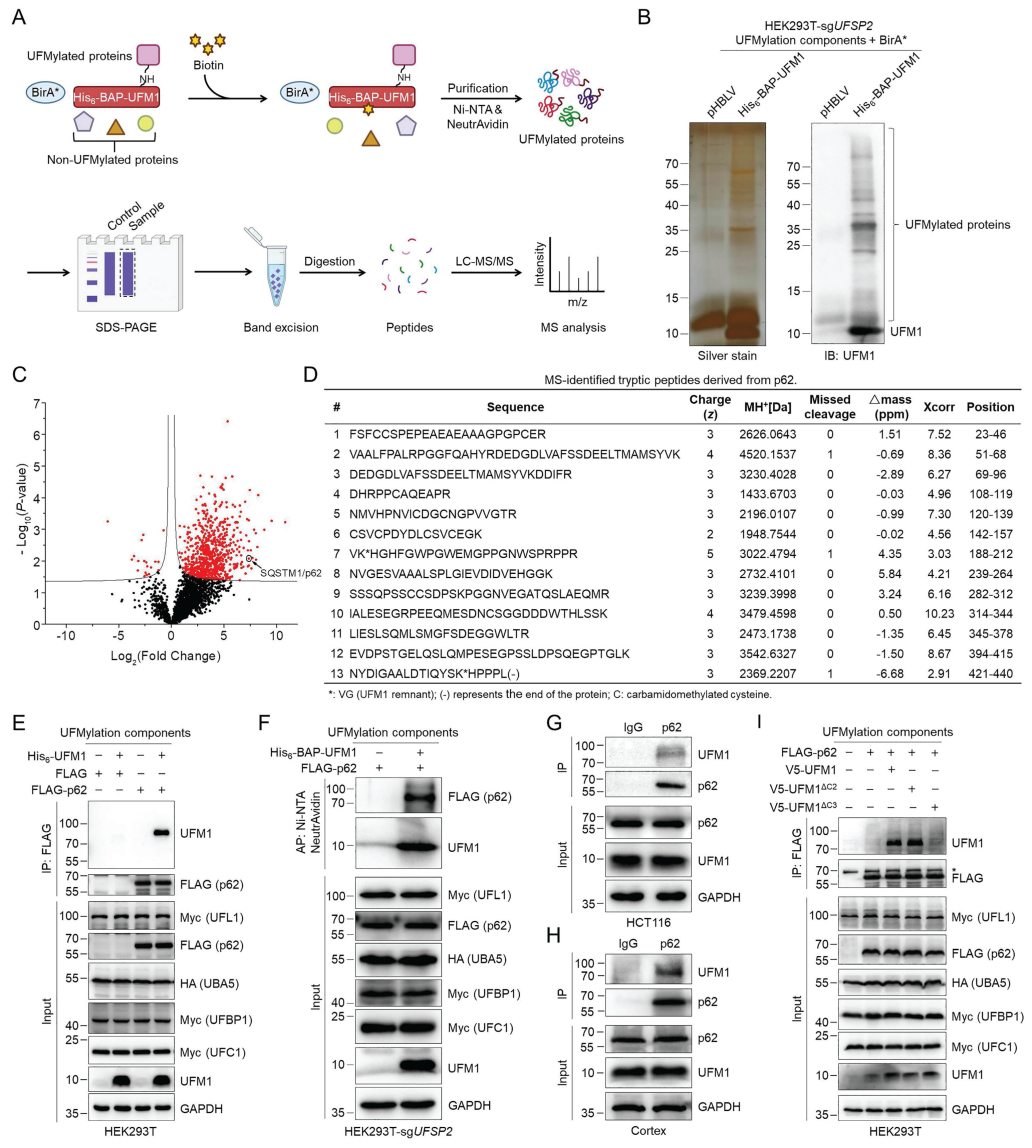
** Identification and verification of p62 UFMylation by quantitative proteomics and biochemical approaches.** (**A**) Schematic illustration of the experimental procedure for the identification of putative UFMylation substrates. HEK293T-sg*UFSP2* cells were transfected with the UFMylation components (including His_6_-BAP-UFM1, HA-UBA5, Myc-UFC1, Myc-UFL1, and Myc-UFBP1) and BirA* for 24 h, and treated with biotin (20 μM) for 24 h. UFMylated proteins were purified from cell lysates sequentially with Ni-NTA resin and NeutrAvidin agarose under denaturing conditions. The purified UFMylated proteins were separated by SDS-PAGE and digested in gel with trypsin. Peptides were desalted with C18 ZipTip and analyzed by LC-MS/MS followed by database search. (**B**) Silver staining and immunoblotting of UFM1 for the affinity-purified proteins. (**C**) A volcano plot for the affinity-purified proteins identified by LC-MS/MS. p62/SQSTM1 was indicated by the open black circle. (**D**) List of MS-identified tryptic peptides derived from p62. The amino acid sequence, charge state (*z*), MH^+^, missed cleavage #, Δmass (ppm), Xcorr, and amino acid position for the peptides were provided. (**E**) Biochemical validation of p62 as a UFMylation substrate. HEK293T cells were transfected with His_6_-UFM1, HA-UBA5, Myc-UFC1, Myc-UFL1, Myc-UFBP1, and/or FLAG-p62 for 48 h. p62 was purified with anti-FLAG affinity gel and eluted with FLAG peptide (200 μg/mL). The cell lysates and purified samples were immunoblotted. (**F**) Tandem affinity purification and immunoblotting of the UFMylated p62. The cell lysates and proteins affinity-purified according to the experimental procedure in Fig. 1A were immunoblotted. (**G-H**) Endogenous p62 is UFMylated. HCT116 cells (G) and mouse cortex (H) were lysed and endogenous p62 was immunoprecipitated with IgG or anti-p62 antibodies for immunoblotting analysis. (**I**) UFMylation assay of p62. HEK293T cells were transfected with FLAG-p62, HA-UBA5, Myc-UFC1, Myc-UFL1, Myc-UFBP1, and V5-UFM1/V5-UFM1^ΔC2^/V5-UFM1^ΔC3^ for 48 h. The UFMylation assay was performed as described in (E). *: non-specific signal.

To investigate whether UBA5 or UFL1 directly modulates the UFMylation of autophagy-related proteins to regulate autophagic flux, we performed tandem affinity purification and quantitative proteomics analysis of possible UFMylated proteins. We expressed the tagged UFMylation components, including His_6_-BAP-UFM1, HA-UBA5, Myc-UFC1, Myc-UFL1, and Myc-UFBP1, and BirA* in HEK293T-sg*UFSP2* cells, followed by biotin treatment and cell lysis. The UFMylated proteins were first purified with Ni-NTA resin and further enriched with NeutrAvidin agarose resin under denaturing conditions (Fig. 2A). The purification was verified by silver staining and Western blotting (Fig. 2B), followed by in-gel trypsinolysis. The resulting peptides were subjected to LC-MS/MS followed by data analysis. The identified proteins from three biological replicates were quantified to generate the volcano plot. Among the 2473 identified proteins (Table S1), 738 proteins (Table S2) exhibited statistically significant differences and were considered putative UFMylated substrates (Fig. 2C). Many previously discovered UFMylated proteins, including UFBP1, RPL26, Histone H4, p53, P4HB, and PARP1 (Table S2), were also identified, indicating the effectiveness of our high-throughput proteomic approach. Interestingly, among them, p62, a critical autophagy receptor 8, is a novel UFMylation candidate. LC-MS/MS confidently identified 13 unique tryptic peptides derived from p62 with a sequence coverage of 71.14%, one of them bearing the UFMylation site (Fig. 2D). We speculate that UFMylation of p62 may play a critical function in modulating autophagy flux.

To verify whether p62 is a target for UFMylation biochemically, we co-expressed FLAG-p62 with the UFMylation components in HEK293T cells and isolated p62 using FLAG affinity gel. Immunoblotting of the purified p62 with an anti-UFM1 antibody detected a band at a molecular weight approximately equal to the sum of p62 and UFM1 (Fig. 2E). To rule out the possible noncovalent interaction of UFMylated proteins, we further immunoblotted the tandem affinity-purified samples obtained under denaturing conditions based on the procedure described in Fig. 2A from cells expressing FLAG-p62 and the UFMylation components. The result explicitly demonstrated that p62 migrated to a higher molecular weight region with the addition of a UFM1 molecule, confirming that UFM1 was covalently conjugated to p62 (Fig. 2F). The anti-UFM1 immunoblotting of immunoprecipitated endogenous p62 from HCT116 cells and mouse cortex also detected a clear band at the similar molecular weight, indicating that the endogenous p62 was indeed UFMylated (Fig. 2G-H). Our experiments further revealed that p62 can be mono-UFMylated via expressing either premature (UFM1) or mature UFM1 (UFM1^ΔC2^), but not the conjugation-defective UFM1 mutant (UFM1^ΔC3^) (Fig. 2I). Based on the above experiments, we could confidently conclude that p62 is indeed a bona fide UFMylation substrate.

**Figure 3:**
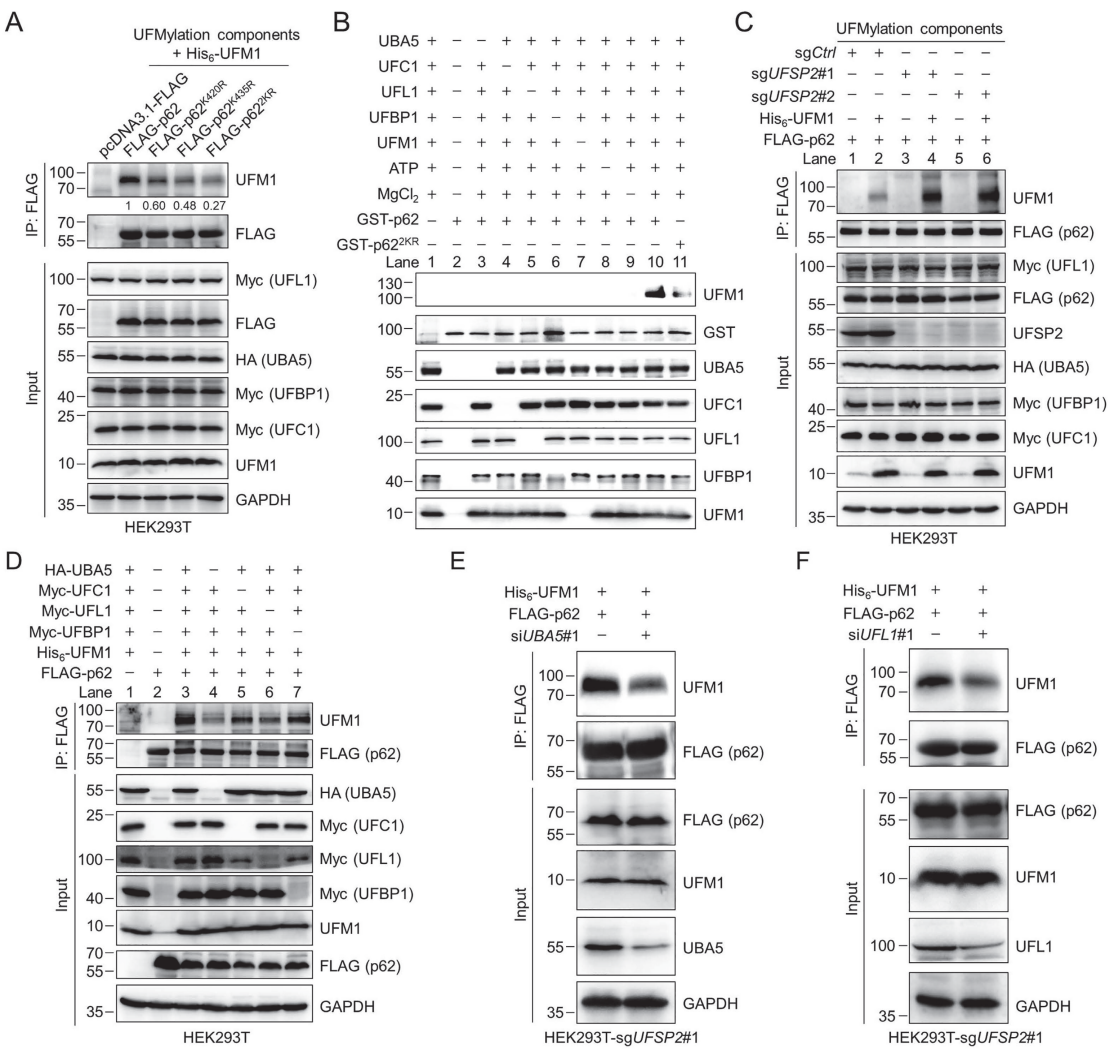
** Biochemical identification of p62 UFMylation sites and knockdown of *UBA5* or *UFL1* attenuates p62 UFMylation.** (**A**) K420 and K435 are the major UFMylation sites on p62. HEK293T cells were transfected with the wild-type (WT) p62 or its K-to-R mutants, enzymes in the UFMylation system, and His_6_-UFM1. The UFMylation assay was performed as described in Fig. 2E. The relative intensity for the p62 UFMylation was depicted below the image. (**B**) *In vitro* p62 UFMylation. Recombinant UFM1 conjugation components (UFM1^ΔC2^, UBA5, UFC1, UFL1, UFBP1) and GST-p62 or GST-p62^2KR^ were incubated in the reaction buffer at 30 °C for 2 h. The reaction was terminated by adding 5 × SDS sample loading buffer and heating at 98 ºC for 10 min, and the samples were immunoblotted with the indicated antibodies. (**C**) *UFSP2* knockout enhances p62 UFMylation. The UFMylation assay was performed in the *UFSP2* knockout HEK293T cells as described in Figure 2E. (**D**) Effect of UBA5, UFC1, UFL1, and UFBP1 on p62 UFMylation. HEK293T cells were transfected with different combinations of the UFM1 conjugation components for 48 h and the UFMylation assay was performed as described in Fig. 2E. (**E-F**) p62 UFMylation is decreased after knocking down *UBA5* (E) or *UFL1* (F). *UFSP2* knockout HEK293T cells were transfected with si*NC*, si*UBA5*#1, or si*UFL1*#1 along with FLAG-p62 and His_6_-UFM1 plasmids for 48 h. p62 was immunoprecipitated with anti-FLAG affinity gel. The cell lysates and immunoprecipitates were immunoblotted.

We next sought to identify the UFMylation sites on p62. Since the quantitative proteomics identified one potential UFMylation site (K435) on p62 with high confidence (Fig. S2), and K435 is located near the C-terminal tail of p62, which usually collaborates with K420 in contributing to the function of the p62 UBA domain, we generated several p62 K-to-R mutants and performed the UFMylation assay. The results demonstrated that the K420R and K435R mutations reduced the p62 UFMylation, and the K420/435R p62 double mutant (referred to as p62^2KR^) can hardly be UFMylated (Fig. 3A). The UFMylation of p62 was further confirmed *in vitro* (Fig. 3B). Purified GST-p62 and GST-p62^2KR^ were incubated in the UFMylation buffer containing UBA5, UFC1, UFL1, UFM1^ΔC2^, ATP, and MgCl_2_. The reaction was stopped by adding SDS sample loading buffer, and the samples were subjected to immunoblotting with anti-UFM1 antibody. The result demonstrated that p62 was clearly UFMylated (lane 10) while p62^2KR^ was barely UFMylated (lane 11), indicating that K420 and K435 are the major UFMylation sites on p62. Additionally, we examined the UFMylation of p62 in cortical tissues from mice at different ages and found that UFMylation of p62 gradually decreased as age increased, suggesting that p62 UFMylation may be age-dependent (Fig. S3A-B).

To further exploit the function of each component in the UFMylation system, we conducted the following experiments. When we overexpressed p62 and the UFMylation components in the HEK293T-sg*UFSP2* cells, the p62 UFMylation level was strikingly elevated compared with the sg*Ctrl* cells (lanes 4 and 6) (Fig. 3C). Furthermore, p62 UFMylation was markedly reduced when UBA5 (lane 4) or UFL1 (lane 6) was not ectopically expressed. However, UFMylated p62 could be readily detected when cells did not express exogenous UFC1 (lane 5) or UFBP1 (lane 7) (Fig. 3D). Notably, after depletion of either *UBA5* or *UFL1* by specific siRNAs, the p62 UFMylation level was diminished (Fig. 3E-F). These results indicate that UBA5 and UFL1 are important, at least under our experimental conditions, for p62 UFMylation, presumably due to their insufficient endogenous expression in this cell line, and UFSP2 can remove the conjugated UFM1 from p62.

### p62 UFMylation modulates autophagosome clearance by elevating autophagic flux

**Figure 4:**
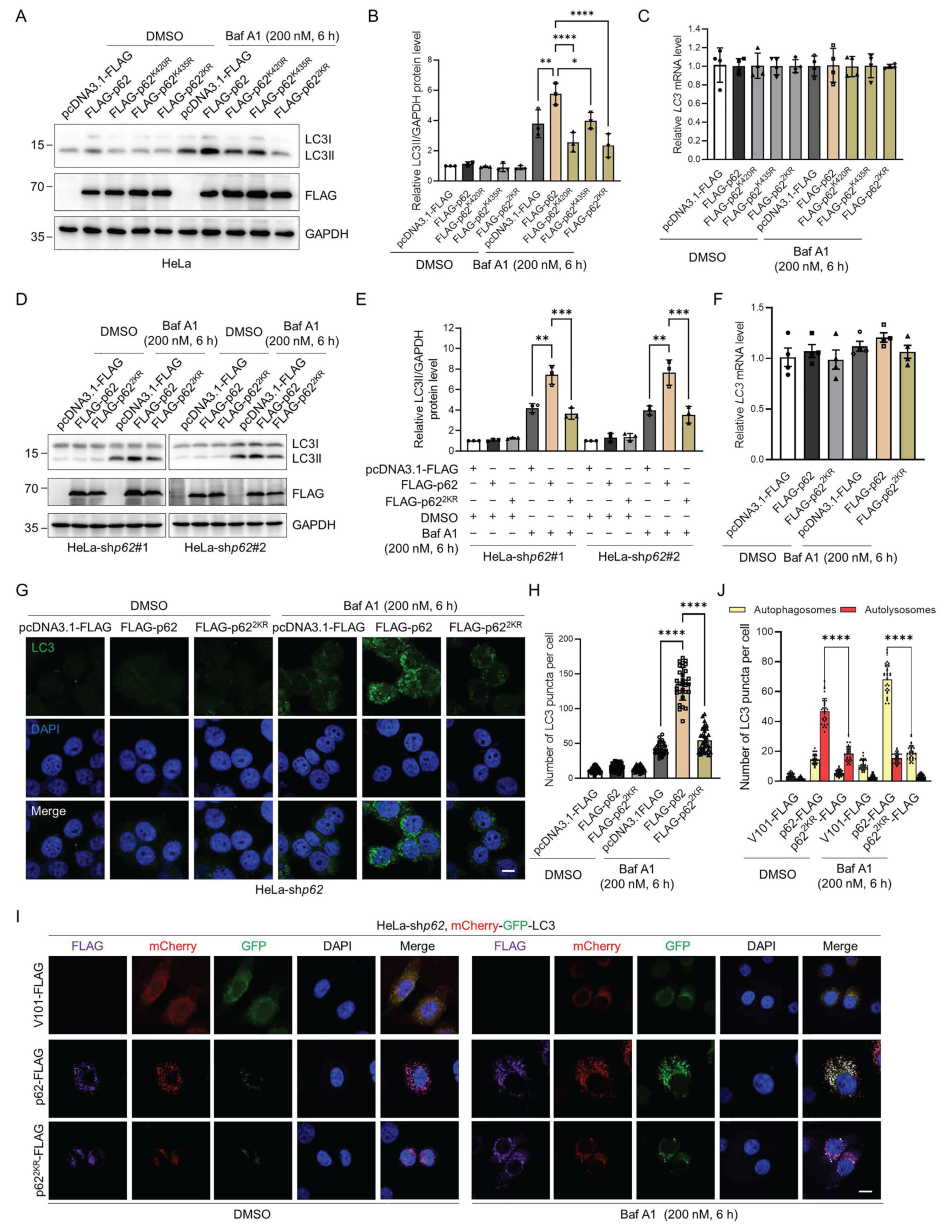
** p62 UFMylation is required for LC3II accumulation in autophagic degradation.** (**A-B**) UFMylation-deficient p62 mutation abolishes the effect of p62 on autophagy. HeLa cells were transfected with pcDNA3.1-FLAG, FLAG-p62, FLAG-p62^K420R^, FLAG-p62^K435R^, or FLAG-p62^2KR^ plasmid for 42 h, and then treated with Baf A1 (200 nM) for 6 h. Cell lysates were subjected to immunoblotting analysis. The relative protein levels of LC3II were normalized to GAPDH and quantified. Mean ± SD (n = 3 biological replicates), two-way ANOVA with Tukey's multiple comparisons post hoc test, *: *P* < 0.05, **: *P* < 0.01, ****: *P* < 0.0001. (**C**) Mutation of the p62 UFMylation sites does not affect the *LC3* mRNA level. Total RNA was isolated from HeLa cells treated as described in (A) and subjected to RT-qPCR analysis. (**D-E**) The absence of UFMylation in p62 eliminates the regulatory role of p62 on autophagy. *p62* knockdown HeLa cells were transfected with pcDNA3.1-FLAG, FLAG-p62, or FLAG-p62^2KR^ plasmid for 42 h, and then treated with Baf A1 (200 nM) for 6 h. Cell lysates were subjected to immunoblotting analysis (D) and quantification (E). The relative protein levels of LC3II were normalized to GAPDH and quantified. Mean ± SD (n = 3 biological replicates), two-way ANOVA with Tukey's multiple comparisons post hoc test, **: *P* < 0.01, ***: *P* < 0.001. (**F**) UFMylation-deficient p62 mutation does not affect the *LC3* mRNA level in *p62* knockdown HeLa cells. Total RNA was isolated from *p62* knockdown HeLa cells treated as described in (D) and subjected to RT-qPCR analysis. Mean ± SD (n = 3 biological replicates), two-way ANOVA with Tukey's multiple comparisons post hoc test. (**G-H**) UFMylation-deficient mutation eliminates the influence of p62 on the formation of LC3 puncta in *p62* knockdown HeLa cells.* p62* knockdown HeLa cells were transfected with pcDNA3.1-FLAG, FLAG-p62, or FLAG-p62^2KR^ plasmid for 42 h, and then treated with Baf A1 (200 nM) for 6 h for immunofluorescence imaging (G) and quantification (H). Scale bar, 10 μm. Mean ± SD (n = 3 biological replicates with 30 cells, each point represented the number of puncta in one cell), two-way ANOVA with Tukey's multiple comparisons post hoc test, ****: *P* < 0.0001. (**I-J**) p62-enhanced autophagic flux is blocked by UFMylation-deficient p62 mutation. *p62* knockdown HeLa cells were transfected with the mCherry-GFP-LC3 plasmid and V101-FLAG, p62-FLAG, or p62^2KR^-FLAG plasmid, and then treated with DMSO or Baf A1 (200 nM) for 6 h for confocal imaging (I) and quantitative analysis (J) of autophagosomes (yellow puncta) and autolysosomes (red puncta). Scale bar, 10 μm. Mean ± SD (n = 3 biological replicates with 30 cells, each point represented the number of puncta in one cell), two-way ANOVA with Tukey's multiple comparisons post hoc test, ****: *P* < 0.0001.

p62 is a critical autophagy receptor and could be modified by an array of PTMs that modulate its autophagic activity 16-22, 47-49. Therefore, we speculated that p62 UFMylation might also affect its autophagic activity. Consistent with this hypothesis, when p62 was overexpressed, LC3II accumulated upon Baf A1 treatment due to the blockage of lysosomal degradation. Strikingly, expression of the UFMylation-deficient p62 mutant significantly attenuated this Baf A1-induced LC3II accumulation without affecting its mRNA level. This indicates that impairment of p62 UFMylation diminishes autophagic flux (Fig. 4A-C and Fig. S4A-C). To exclude the impact of endogenous p62, we examined the function of p62 UFMylation in *p62* knockdown HeLa cells (Fig. S4D). Consistent with the above results, WT p62 but not UFMylation-deficient p62 promoted LC3 accumulation, whereas the *LC3* mRNA level was not altered by p62 mutation (Fig. 4D-F). Additionally, immunostaining of LC3 puncta further confirmed this finding. As expected, WT p62 elevated while p62^2KR^ failed to increase the number of LC3II puncta in *p62* knockdown HeLa cells treated with Baf A1 (Fig. 4G-H). To further evaluate the autophagic flux level, we used tandem-tagged mCherry-GFP-LC3 50 to distinguish autophagosomes and autolysosomes. After autophagosomes fuse with lysosomes, autolysosomes become acidic, leading to the reduction of the GFP fluorescence signal due to its reduced sensitivity to low pH, and thus, only the mCherry (red) fluorescence signal could be detected. Furthermore, Baf A1 treatment resulted in the detection of mCherry-GFP (yellow), which was due to the blockage of the autophagosome-lysosome fusion. Although Baf A1 treatment may increase lysosomal pH and partially restore GFP signal, the results were obtained based on the relative changes under identical experimental conditions. To further validate the results, we overexpressed the WT or UFMylation-deficient p62 mutant p62^2KR^ in *p62* knockdown HeLa cells and examined the intracellular autophagic flux upon DMSO or Baf A1 treatment. Detailed analysis revealed that the number of autolysosomes (red) was markedly increased in cells expressing the WT p62, but it was only marginally increased in the p62^2KR^ expressing cells upon DMSO treatment. Moreover, autophagosomes (yellow) were significantly elevated in cells expressing the WT p62 but not in cells expressing p62^2KR^ in response to Baf A1 (Fig. 4I-J, and Fig. S4E). Taken together, these data suggest that p62 UFMylation modulates autophagosome formation and thus elevates autophagic flux.

**Figure 5:**
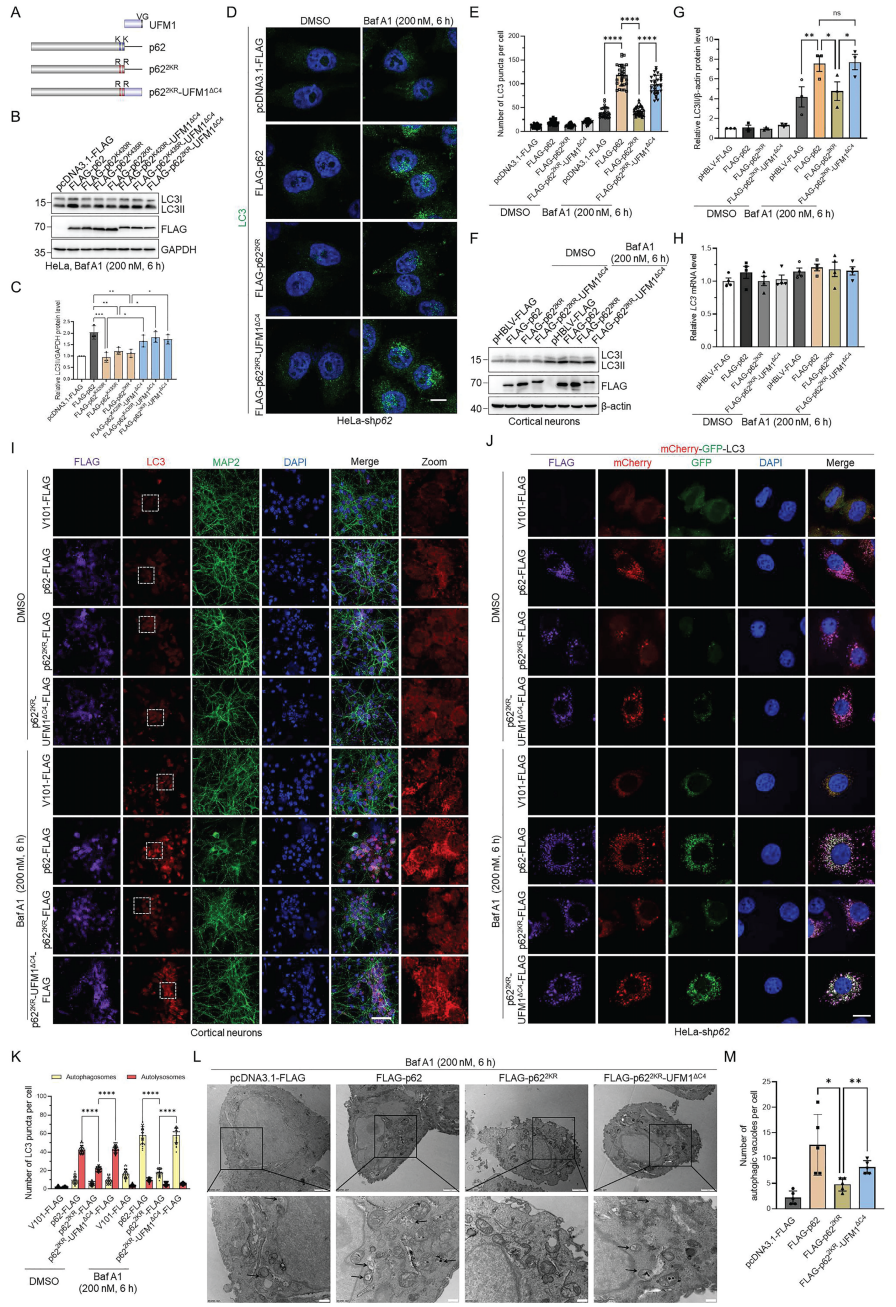
** p62 UFMylation modulates autophagosome clearance by elevating autophagic flux.** (**A**) Schematic representation of UFM1, p62, p62^2KR^, and p62^2KR^-UFM1^ΔC4^. UFM1^ΔC4^ was fused to the C-terminus of p62^2KR^ to mimic the UFMylated p62 because the major UFMylation sites were at the C-terminus of p62. (**B-C**) p62 UFMylation elevates LC3II protein level. HeLa cells were transfected with pcDNA3.1-FLAG, FLAG-p62, FLAG-p62^K420R^, FLAG-p62^K435R^, FLAG-p62^2KR^, FLAG-p62^K420R^-UFM1^ΔC4^, FLAG-p62^K435R^-UFM1^ΔC4^, or FLAG-p62^2KR^-UFM1^ΔC4^ plasmid, and then treated with Baf A1 (200 nM) for 6 h. Cell lysates were subjected to immunoblotting analysis. The relative protein levels of LC3II were normalized to GAPDH and quantified. Mean ± SD (n = 3 biological replicates), one-way ANOVA with Tukey's multiple comparisons post hoc test, *: *P* < 0.05, **: *P* < 0.01, ***: *P* < 0.001. (**D-E**) p62 UFMylation increases LC3 puncta. *p62* knockdown HeLa cells were transfected with pcDNA3.1-FLAG, FLAG-p62, FLAG-p62^2KR^, or FLAG-p62^2KR^-UFM1^ΔC4^ plasmid, and then treated with Baf A1 (200 nM) for 6 h. Cells were immunostained with LC3 and DAPI (D) for quantification (E). Scale bar, 10 μm. Mean ± SD (n = 3 biological replicates with 30 cells, each point represented the number of puncta in one cell), two-way ANOVA with Tukey's multiple comparisons post hoc test, ****: *P* < 0.0001. (**F-G**) p62 UFMylation elevates LC3II protein level in primary cortical neurons. Primary cortical neurons were cultured with conditioned medium for 5 days and infected with pHBLV-FLAG, p62-FLAG, p62^2KR^-FLAG, or p62^2KR^-UFM1^ΔC4^-FLAG lentiviral particles for 42 h, and then treated with DMSO or Baf A1 (200 nM) for 6 h. Cell lysates were subjected to immunoblotting analysis (F) and quantification (G). The relative protein levels of LC3II were normalized to β-actin and quantified. Mean ± SD (n = 3 biological replicates), two-way ANOVA with Tukey's multiple comparisons post hoc test, *: *P* < 0.05, **: *P* < 0.01, ns: not significant. (**H**) UFMylation-deficient p62 mutation does not affect the *LC3* mRNA level in primary cortical neurons. Total RNA was isolated from primary cortical neurons treated as described in (F) and subjected to RT-qPCR analysis. Mean ± SD (n = 4 biological replicates), two-way ANOVA with Tukey's multiple comparisons post hoc test. (**I**) UFMylation-deficient mutation abolishes the effect of p62 on autophagy in primary cortical neurons. Primary cortical neurons were infected with V101-FLAG, p62-FLAG, p62^2KR^-FLAG, or p62^2KR^-UFM1^ΔC4^-FLAG lentiviral particles for 42 h, treated with DMSO or Baf A1 (200 nM) for 6 h, and then stained with FLAG, LC3, MAP2, and DAPI. Scale bar, 50 μm. (**J-K**) p62 UFMylation enhances autophagic flux. *p62* knockdown HeLa cells were transfected with the mCherry-GFP-LC3 plasmid and V101-FLAG, p62-FLAG, p62^2KR^-FLAG, or p62^2KR^-UFM1^ΔC4^-FLAG plasmid for 42 h and then treated with Baf A1 (200 nM) for 6 h for confocal imaging (J) and quantitative analysis (K) of autophagosomes (yellow puncta) and autolysosomes (red puncta). Scale bar, 10 μm. Mean ± SD (n = 3 biological replicates with 30 cells, each point represented the number of puncta in one cell), two-way ANOVA with Tukey's multiple comparisons post hoc test, ****: *P* < 0.0001. (**L-M**) p62 UFMylation partially restores the number of autophagic vacuoles. Transmission electron microscopy was carried out for cells transfected with pcDNA3.1-FLAG, FLAG-p62, FLAG-p62^2KR^, or FLAG-p62^2KR^-UFM1^ΔC4^ plasmid and treated with Baf A1 (200 nM) for 6 h. Representative autophagic vacuoles were indicated by arrows (L) and quantified (M). Scale bar, 500 nm. Mean ± SD (n = 5 biological replicates), one-way ANOVA with Tukey's multiple comparisons post hoc test, *: *P* < 0.05, **: *P* < 0.01.

Since p62^2KR^ exhibited different autophagic activity compared with WT p62, we hypothesized that the regulatory function of p62 might be highly influenced by UFMylation. To further investigate the function of p62 UFMylation, we constructed an artificial p62^2KR^-UFM1^ΔC4^ mutant to mimic p62 UFMylation by fusing UFM1^ΔC4^ (UFM1 with deleted C-terminal 4 amino acids VGSC) to the C-terminus of p62^2KR^, because the major UFMylation sites on p62 are close to its C-terminus (Fig. 5A). This strategy was previously used to study the function of ISGylation and SUMOylation 51, 52. Immunoblotting experiments revealed that LC3II accumulation was increased when WT p62 or p62^2KR^-UFM1^ΔC4^, but not p62^2KR^, was expressed in HeLa cells, followed by Baf A1 treatment (Fig. 5B-C). As expected, immunostaining unveiled that LC3 puncta exhibited similar phenomena as the immunoblotting result, i.e., expression of p62^2KR^-UFM1^ΔC4^ significantly restored the number of LC3 puncta in *p62* knockdown HeLa cells in the presence of Baf A1 (Fig. 5D-E). We then exploited the effect of p62 UFMylation on LC3 puncta in primary cortical neurons. Unsurprisingly, immunoblotting and immunofluorescence experiments uncovered that expression of p62 or p62^2KR^-UFM1^ΔC4^ but not p62^2KR^ recovered LC3II accumulation (Fig. 5F-I) in primary cortical neurons.

In addition, autophagic flux was blocked upon expressing p62^2KR^, whereas p62^2KR^-UFM1^ΔC4^ notably restored the autophagosome formation, indicating that p62 UFMylation enhanced autophagic flux in *p62* knockdown HeLa cells (Fig. 5J-K and Fig. S4F). In accordance with this, transmission electron microscopy disclosed that p62 UFMylation resulted in a partial restoration of autophagosomes (Fig. 5L-M). Cumulatively, these data underscore that p62 UFMylation plays a crucial role in regulating p62-mediated autophagy.

### p62 UFMylation promotes the p62-LC3II interaction and the degradation of pathogenic mHTT

**Figure 6:**
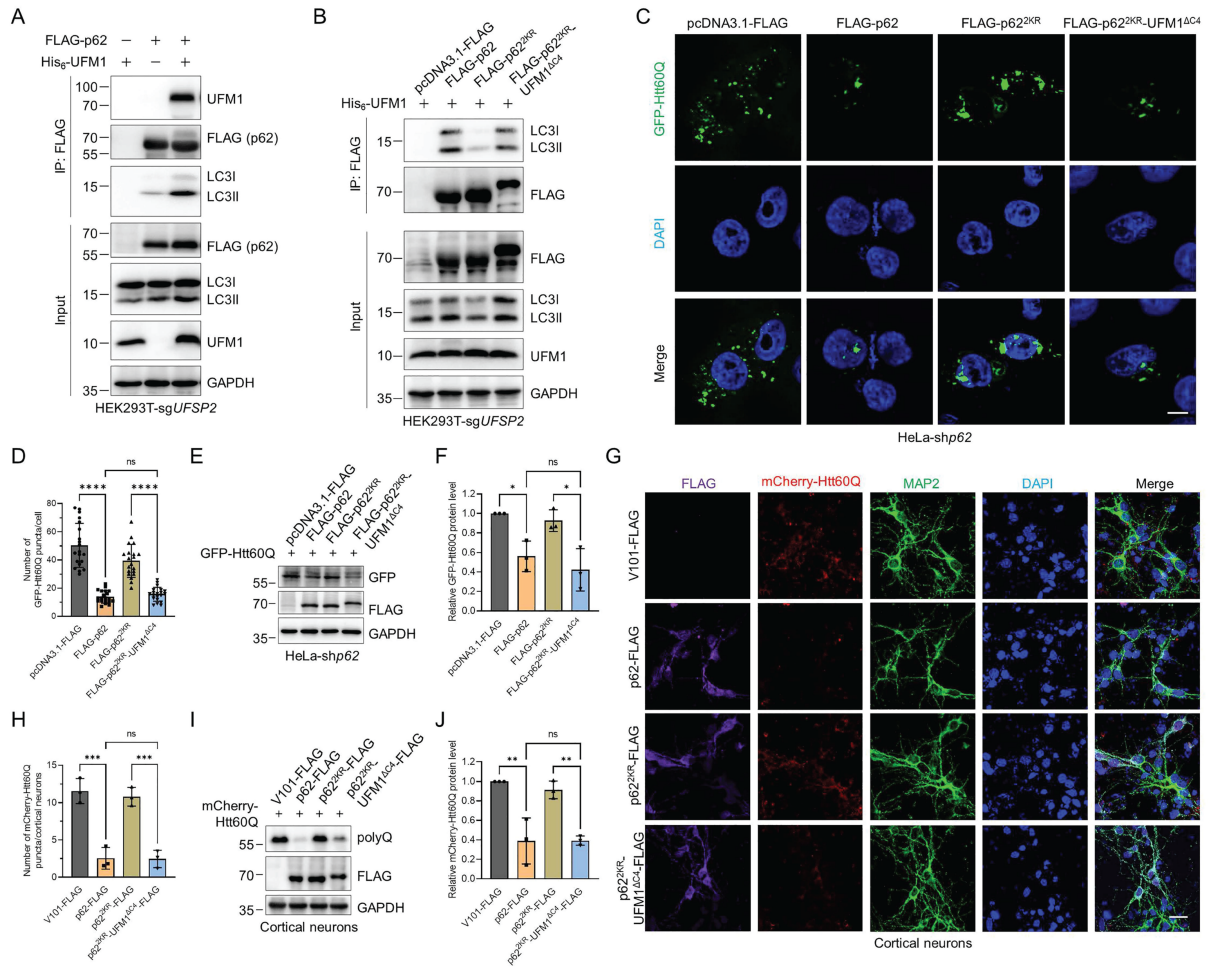
** p62 UFMylation promotes the interaction between p62 and LC3 and enhances the degradation of pathogenic mutant huntingtin (mHTT).** (**A-B**) p62 UFMylation enhances the interaction between p62 and LC3. HEK293T-sg*UFSP2* cells were transfected with FLAG-p62 and/or His_6_-UFM1 plasmids (A) or with pcDNA3.1-FLAG, FLAG-p62, FLAG-p62^2KR^, or FLAG-p62^2KR^-UFM1^ΔC4^ and His_6_-UFM1 plasmids (B) for 48 h. Cells were lysed and p62 was purified with the anti-FLAG antibody. Cell lysates and affinity-purified samples were immunoblotted. (**C-F**) p62 UFMylation promotes the degradation of pathogenic mHTT. *p62* knockdown HeLa cells were transfected with pcDNA3.1-FLAG, FLAG-p62, FLAG-p62^2KR^ or FLAG-p62^2KR^-UFM1^ΔC4^, and GFP-Htt60Q plasmids for 48 h. Representative confocal images (C) were captured for quantification of GFP-Htt60Q (D). The cell lysates were immunoblotted with the indicated antibodies (E) for quantitative analysis of GFP-Htt60Q protein (F). Scale bar, 10 μm. Mean ± SD (n = 3 biological replicates with 20 cells, each point represented the number of puncta in one cell), one-way ANOVA with Tukey's multiple comparisons post hoc test, *: *P* < 0.05, ****: *P* < 0.0001, ns: not significant. (**G-J**) p62 UFMylation promotes the degradation of pathogenic mHTT in primary cortical neurons. Primary cortical neurons were infected with mCherry-Htt60Q and V101-FLAG, p62-FLAG, p62^2KR^-FLAG, or p62^2KR^-UFM1^ΔC4^-FLAG lentiviral particles for 48 h for staining of FLAG, MAP2, and DAPI in primary cortical neurons (G) and quantification of mCherry-Htt60Q (H). Lysates from primary cortical neurons were immunoblotted with the indicated antibodies (I) for quantitative analysis of mCherry-Htt60Q protein (J). Scale bar, 20 μm. Mean ± SD (n = 3), one-way ANOVA with Tukey's multiple comparisons post hoc test, **: *P* < 0.01, ***: *P* < 0.001, ns: not significant.

In above experiments, we observed an intriguing phenomenon that p62 UFMylation modulates autophagosome formation and thus elevates autophagic flux. To investigate the molecular mechanisms by which p62 UFMylation modulates its biological functions, we performed co-immunoprecipitation experiments. We expressed His_6_-Ub and/or UFM1 with FLAG-p62 or GFP-p62 in the *UFSP2* knockout HEK293T cells and then examined the self-oligomerization of p62 and its association with ubiquitinated proteins, given that both p62 dimerization and its binding to ubiquitinated cargos are critical for autophagic flux. Surprisingly, the results revealed that UFM1 affected neither the dimerization of p62 nor its interaction with ubiquitinated proteins (Fig. S5A-B). However, unexpectedly, expression of UFM1 increased the p62 UFMylation and promoted the binding between p62 and LC3 (Fig. 6A). In contrast, p62^2KR^ weakened the interaction between p62 and LC3 while p62^2KR^-UFM1^ΔC4^ restored this interaction (Fig. 6B). Along with the above data, these results clearly suggest that UFMylation enhances p62 autophagic activity by increasing the binding of p62 to LC3.

The finding that p62 UFMylation increased the p62-LC3 interaction suggests a regulatory role of UFMylation in autophagic degradation. Therefore, we tested whether p62 UFMylation affects the degradation of neurodegeneration-associated proteins such as pathogenic HTT mutant GFP-Htt60Q, which fuses a GFP to the N-terminal 1-208 amino acids of HTT with a 60-glutamine tail 53. The results revealed that WT p62, but not p62^2KR^, induced autophagic degradation of Htt60Q. Consistently, the p62 UFMylation mimetic variant p62^2KR^-UFM1^ΔC4^ also enhanced the degradation of Htt60Q in *p62* knockdown HeLa cells (Fig. 6C-F) and primary cortical neurons (Fig. 6G-J). Additionally, overexpression of p62 or its UFMylation mimetic variant p62^2KR^-UFM1^ΔC4^ promoted the colocalization of LC3 with Htt60Q. Conversely, expression of p62^2KR^ reduced their colocalization (Fig. S6A-C). To determine whether the UFMylation of p62 specifically regulates substrate degradation, we examined the interaction between GFP-Htt60Q and WT p62, p62^2KR^, or p62^2KR^-UFM1^ΔC4^. Immunoprecipitation and immunoblotting experiments revealed that the UFMylation of p62 did not alter its binding affinity for GFP-Htt60Q (Fig. S7A). Consistently, both WT p62 and the p62^2KR^-UFM1^ΔC4^ promoted the degradation of other neurodegeneration-associated proteins, such as GFP-Tau^P301L^, RFP-SOD1^G85R^, RFP-SOD1^G93A^, whereas the p62^2KR^ did not affect their degradation (Fig. S7B-D). These data demonstrate that UFMylation increases the interaction between p62 and LC3 and promotes the autophagic degradation of neurodegeneration-associated proteins.

### p62 UFMylation promotes the degradation of pathogenic mHTT in HD mice

**Figure 7:**
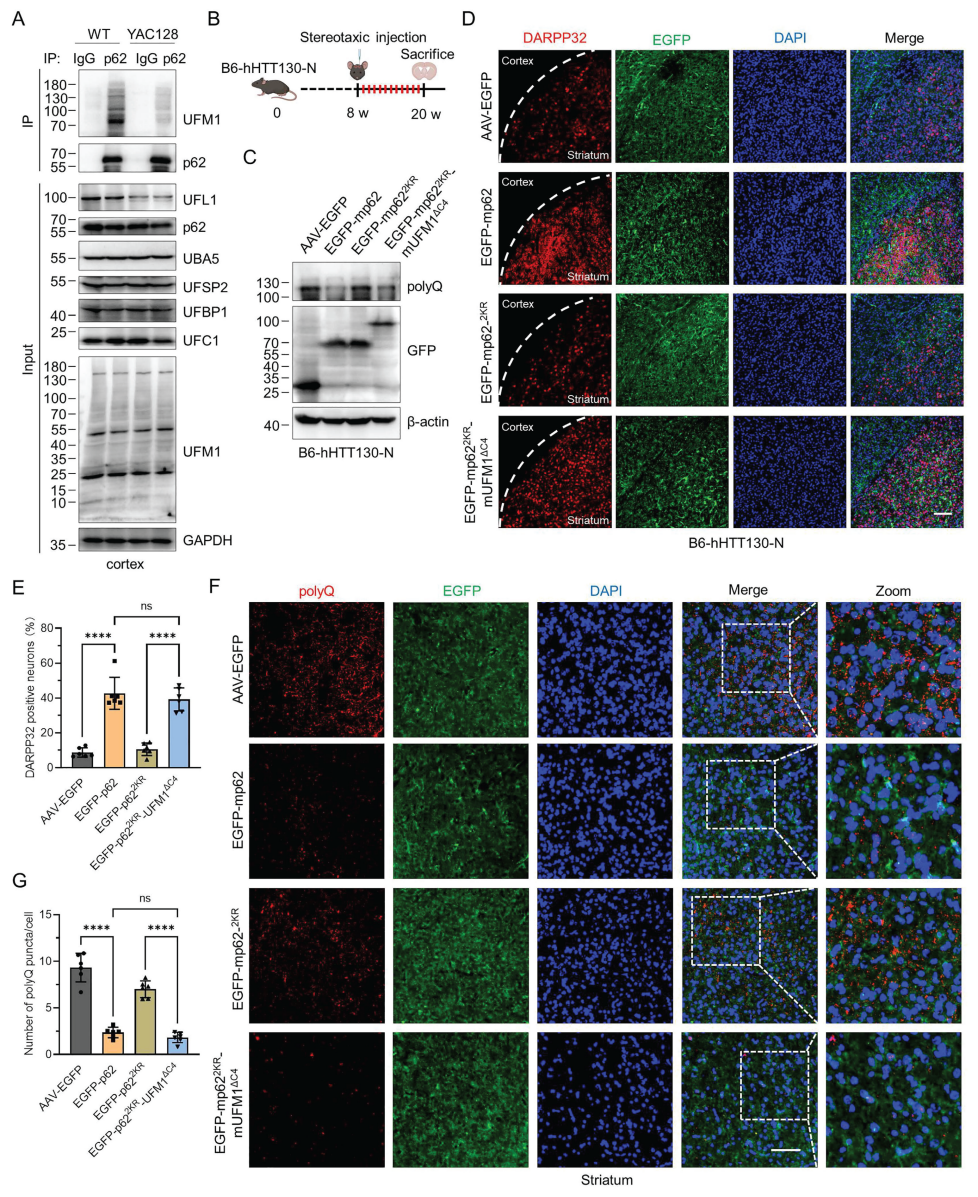
** p62 UFMylation enhances the degradation of pathogenic mHTT in the cortex of HD mice.** (**A**) p62 UFMylation is decreased in the cortex of HD mice. The cortex of WT or YAC128 mice was lysed and endogenous p62 was immunoprecipitated with IgG or anti-p62 antibodies for immunoblotting analysis. (**B**) Schematic illustration of stereotaxic viral injection. B6HTT-CAG130-N mice (8 weeks) were stereotaxically injected with adeno-associated virus in the striatum (coordinates anteroposterior = -1.0 mm, mediolateral = ±2.0 mm, dorsoventral = -3.3 mm). At 12 weeks after injection, the mice were anesthetized and perfused with ice-cold PBS and 4% paraformaldehyde for brain isolation. (**C**) p62 UFMylation promotes the degradation of mHTT protein in HD mice. At 12 weeks after injection of the indicated adeno-associated virus, the striata of mice were collected and lysed for immunoblotting. (**D-E**) p62 UFMylation increases the density of medium spiny neurons (MSNs). The mouse brains from (B) were cut into 20 μm sections with a freezing microtome, and the slices were immunostained with DARPP32 and DAPI. Representative confocal images were captured using a confocal microscope (D) for quantification (E) of DARPP32-positive neurons. Scale bar, 100 μm. Mean ± SD (n = 6), one-way ANOVA with Tukey's multiple comparisons post hoc test, ****: *P* < 0.0001, ns: not significant. (**F-G**) p62 UFMylation promotes the degradation of mHTT protein in the HD mouse striatum. The mouse brain slices from (D) were immunostained with anti-polyQ antibody and DAPI. Images were scanned using a confocal microscope (F) for quantification (G) of positive polyQ staining. Scale bar, 30 μm. Mean ± SD (n = 6), one-way ANOVA with Tukey's multiple comparisons post hoc test, ****: *P* < 0.0001, ns: not significant.

To comprehensively evaluate the regulatory function of p62 UFMylation in the pathogenesis of HD, we assessed the UFMylation levels of endogenous p62 in the YAC128 and B6-hHTT130-N transgenic mice 54-57. Affinity purification and immunoblotting results disclosed a significant decline in p62 UFMylation in the cortex of HD mice compared to the WT mice (Fig. 7A and Fig. S8). We subsequently sought to determine whether p62 UFMylation modulates the clearance of mHTT protein in the B6-hHTT130-N mouse striatum. The B6-hHTT130-N mouse carries an N-terminal fragment of the human *HTT* gene with a 130 CAG repeat expansion, and has been extensively used to study mHTT turnover and degradation mechanisms.

This is because the fragment represents key toxic species implicated in HD pathogenesis and is particularly amenable to assessing alterations in mHTT clearance over relatively short experimental time frames 56. We generated three distinct murine p62 constructs: FLAG-mp62, UFMylation-deficient mutant FLAG-mp62^2KR^ (K422R and K437R), and UFMylation mimetic variant FLAG-mp62^2KR^-mUFM1^ΔC4^. Immunoblotting coupled with immunoprecipitation confirmed the protein expression and UFMylation of WT and mutant murine p62 (Fig. S9A-B). Then we employed a viral stereotactic injection of recombinant adenoviruses encoding these constructs into the striatum of B6-hHTT130-N mice (Fig. 7B). Analysis of striatal lysate demonstrated that UFMylation deficiency did not promote the clearance of mHTT, whereas mp62 UFMylation mimetic variant decreased the mHTT levels (Fig. 7C). Immunofluorescence results further established that p62 UFMylation preserved the density of MSNs (Fig. 7D-E). In addition, the positive polyQ staining was significantly decreased in the striatum of both AAV9-mp62 and AAV9-mp62^2KR^-mUFM1^ΔC4^, indicating that p62 UFMylation is capable of augmenting the degradation of mHTT in the striatum of HD mice (Fig. 7F-G). Collectively, these findings demonstrate that UFMylation serves as a critical PTM for p62, which facilitates the clearance of pathogenic mHTT in HD mice.

## Discussion

**Figure 8:**
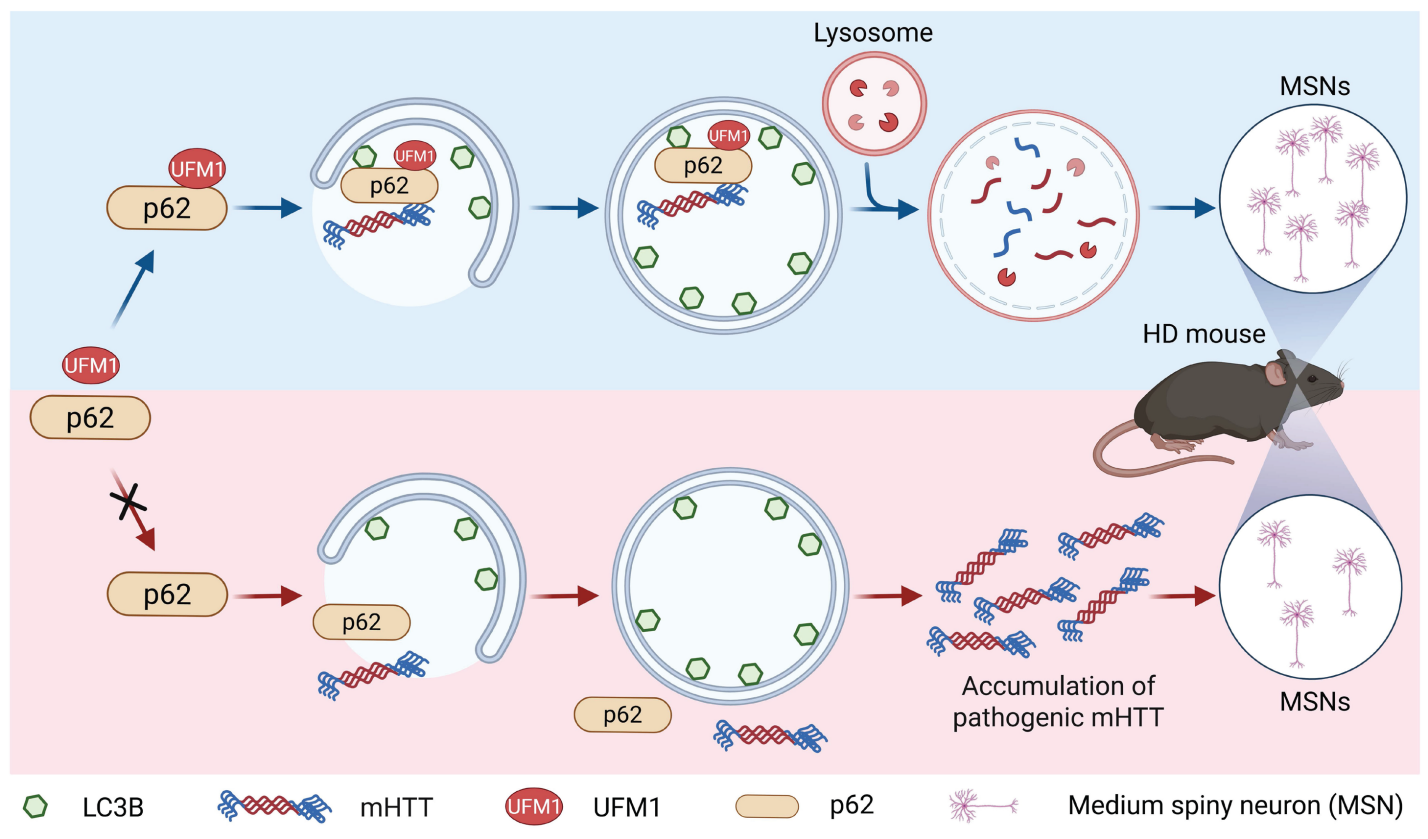
** A proposed model for the regulation of autophagic degradation by p62 UFMylation.** UFMylation of p62 enhances autophagy by promoting its interaction with LC3. In a Huntington's disease mouse model, p62 facilitates the degradation of mutant huntingtin (mHTT) protein via UFMylation and increases the number of medium spiny neurons (MSNs) in the striatum.

The autophagy-lysosome pathway is the major pathway to remove long-lived proteins, insoluble aggregates, stress granules, and dysfunctional organelles within eukaryotic cells 58. In this pathway, p62 is a classical receptor that recruits cargos to form autophagosomes and subsequently fuses with lysosomes for degradation 8. The function of p62 could be precisely controlled by phosphorylation, acetylation, ubiquitination, and FAT10ylation in response to a number of intracellular stimuli 16-22, 47-49. Here, our affinity purification and quantitative proteomics identifies p62 as a bona fide substrate of UFMylation, which substantially expands the pleiotropic functions of p62 on autophagy. Biochemical experiments discovered that two UFMylation components, UBA5 and UFL1, were important for p62 UFMylation and autophagic degradation. Mapping of the modification sites revealed that K420 and K435 were two major UFMylation sites on human p62. Deficiency of UFMylation on p62 (p62^2KR^) abolished the enhanced effect of p62 on autophagic degradation, while fusion of UFM1^ΔC4^ to p62^2KR^ (p62^2KR^-UFM1^ΔC4^) significantly recovered the function of p62 upon autophagic inhibition. Mechanistic studies demonstrated that p62 UFMylation affected neither its dimerization nor its recruitment of ubiquitinated proteins. Instead, p62 UFMylation enhanced the binding of p62 to LC3, thereby promoting the autophagic degradation of pathogenic mHTT. Meanwhile, p62 UFMylation was shown to promote autophagic flux and autophagic degradation of mHTT in mouse primary cortical neurons and the striatum of HD mice. Taken together, our work demonstrated that UFMylation of p62 at specific sites promoted its interaction with LC3 and thus recruited mHTT to form mature autophagolysosomes for their degradation. However, disruption of p62 UFMylation impairs the maturation of autolysosomes, resulting in the accumulation of empty autophagolysosomes. Consequently, pathogenic proteins are accumulated, leading to dysfunction in the degradation of cargo proteins or damaged organelles through the ALP (Fig. 8). Coincidently, our proposed mechanism aligns with the observation of empty autophagolysosomes in the brains of HD patients and YAC128 mice, providing a mechanistic explanation for previously reported p62-associated autophagic defects in HD 12, 14.

Accumulating evidence indicates that impairment of selective autophagy is highly correlated with the development of neurodegenerative diseases and that enhancing this pathway can confer neuroprotective effects 5, 59. Recent studies have demonstrated that pharmacological strategies designed to selectively degrade mHTT by autophagy alleviate disease phenotypes in HD mouse models 7. These findings underscore the importance of efficient cargo recognition and delivery in selective autophagy during neurodegeneration. Consistent with this concept, our study identifies UFMylation of p62 as a regulatory modification that facilitates the clearance of mHTT. Notably, we observed a reduction in p62 UFMylation levels in the cortex of HD model mice, suggesting that dysregulation of UFMylation may be linked to disease-associated defects in selective autophagy. From a mechanistic perspective, we further show that expression of a p62 UFMylation-mimetic variant significantly decreases the accumulation of polyQ in the mouse striatum, supporting the function of p62 UFMylation in limiting the formation of toxic protein aggregation *in vivo*. UFMylated p62 may reduce mHTT aggregates by degrading non-aggregated mHTT. However, how UFMylation of p62 enhances aggregate clearance and functional improvement in animals remains to be further elucidated. In addition, our results indicate that p62 UFMylation broadly enhances the clearance of diverse aggregation-prone proteins, suggesting that it upregulates autophagic flux rather than acting in a cargo-specific manner.

Although previous genetic screens revealed that components in the UFMylation system regulate autophagy 42-45, it is unknown whether any proteins in the ALP are modified by UFM1 and, if so, what the functions of the modified proteins are. Our quantitative proteomics identified many putatively modified proteins, including previously identified UFMylated proteins such as Histone H4 33, P4HB 60, p53 36, and RPL26 31. Although tandem MS did not detect modification sites for most proteins, UFMylation sites were identified for RPL26 and all the components in the UFMylation system, such as UBA5, UFC1, UFL1, and UFBP1 (Table S3). Detection of multiple UFMylation sites on UFM1, UFC1, and UFBP1 or two modification sites on one peptide indicates the presence of possible polyUFMylation or multi-mono-UFMylation. These results also indicate that the MS-identified proteins are bona fide or putatively UFMylated proteins. The following reasons might prevent us from identifying the modification sites for the majority of modified proteins. First, some peptides containing UFM1 remnant (Val-Gly) may be too long or too short to be confidently identified by tandem MS. Second, the modified peptides account for only a small fraction of the total peptides analyzed by tandem MS because only one or a few sites in one protein are UFMylated. In this case, the efficiency for the identification of UFMylation sites might be improved if peptides containing the UFM1 remnant could be selectively enriched.

Our work identified the putative UFMylated proteins in cells overexpressing enzymes in the UFM1-conjugation system and biochemically validated the p62 UFMylation. We also discovered the UFMylation of endogenous p62 in cell lines and the mouse cortex. However, the modification patterns were slightly different in these two systems. In human cell lines, we only detected mono-UFMylation for WT p62. It is also possible that the level of polyUFMylation or multi-mono-UFMylation of p62 in these cell lines is much lower than that of the mono-UFMylation, preventing their clear detection on the Western blotting image. However, we detected multiple UFMylated bands after immunoprecipitation of p62 from the mouse cortical tissue although the top bands were much weaker, indicating the presence of multi-mono-UFMylation or polyUFMylation on p62. While we could not tell the exact type of UFMylation for p62 in the mouse cortical tissue, we observed decreased signal for the modified bands in the cortex of aged mice, implying that the UFM1-conjugation system may be dysfunctional in the tissue of aged animals, which is consistent with our discovery that impairment of p62 UFMylation accumulates pathogenic proteins. These data also suggest that the UFM1-conjugation system might vary in cell types, tissues, and organisms.

Although our tandem MS identified p62 K420 bearing the UFM1 remnant (Val-Gly), our biochemical experiments demonstrated that K420 and K435 were the major UFMylation sites. It is likely that a mutation on one site may affect the modification of other sites. This is consistent with the fact that the position for the remaining weak UFMylation of p62 on Western blot after mutating K420 and K435 to Arg is the same as the UFMylated WT p62. These data also indicate that p62 is most likely mono-UFMylated in cells. Although we have identified the major UFMylation sites on p62, it is possible that other modification sites were not detected or detectable by the tandem MS procedure used in this study due to the weak signal or extreme length of the resulting tryptic peptides.

In our experiments, we mutated the major UFMylation sites on p62 to obtain the UFMylation-deficient p62 mutant (p62^2KR^) and then fused the UFM1 with the deleted C-terminal 4 amino acids to this mutant (p62^2KR^-UFM1^ΔC4^) to mimic the UFMylated p62. Our experiments demonstrated that p62^2KR^ lost the function of WT p62 whereas p62^2KR^-UFM1^ΔC4^ partially or nearly completely recovered the function of WT p62, indicating that p62 UFMylation significantly contributes to the autophagic activity. It should be mentioned that p62^2KR^-UFM1^ΔC4^ may not completely restore the function of p62 UFMylation for the following possible reasons. First, the p62^2KR^-UFM1^ΔC4^ fusion protein is not exactly the same as the UFMylated p62 because the chain topology is different from the UFMylated p62 although the major UFMylation sites are located at the p62 C-terminus. Second, UFMylation is a reversible modification that could be eliminated by UFM1-specific proteases. However, p62^2KR^-UFM1^ΔC4^ cannot be deUFMylated. Third, the identified major UFMylation sites on p62 could also be acetylated or ubiquitinated 18, 21. Therefore, we could not completely rule out their contribution and the crosstalk between these PTMs and UFMylation on the regulation of autophagic activity. However, it has been demonstrated that the p62^2KR^ mutation did not affect the ubiquitination of p62 18. Nevertheless, this is one of the best strategies to explore the function of p62 UFMylation.

The activity of p62 is tightly regulated by diverse PTMs. Phosphorylation of p62 at Ser403 by casein kinase 2 and TANK-binding kinase 1 strengthens the interaction between p62 and K63-linked polyubiquitin chains. This modification promotes the formation of sequestosome, thereby enhancing the autophagic clearance of pathogenic protein aggregates 16, 17. It has been demonstrated that ubiquitination of the UBA domain represents a key regulatory mechanism that determines the binding affinity of p62 for ubiquitinated proteins. However, the p62 UBA domain has a low affinity for ubiquitin and this interaction is further attenuated by its homodimerization 9. The Keap1/Cullin-3/RBX1 E3 ubiquitin ligase complex mediates the ubiquitination of p62 at K420 in the UBA domain, thereby increasing the interaction between p62 and LC3, enhancing its sequestering activity, and promoting the p62-mediated autophagic degradation 19. In addition, the ubiquitin-specific protease USP8 selectively removes ubiquitin from K420 on p62 and consequently attenuates the autophagic flux 49. Moreover, acetylation modulates the autophagic activity of p62. Mechanistically, p62 acetylation at K420 and K435 impairs the UBA domain homodimerization and enhances its affinity to ubiquitin, but does not affect the affinity of p62 to LC3, thus inducing the p62 body formation and its autophagic degradation and contributing to cell survival under nutrient stress 18. Our experiments revealed that UFM1 failed to affect either p62 dimerization or its association with ubiquitinated proteins. Notably, expression of UFM1 increased the p62 UFMylation and promoted its binding to LC3. In contrast, p62^2KR^ weakened its interaction with LC3 while p62^2KR^-UFM1^ΔC4^ restored this interaction. This differs from the acetylation at the same site on p62, a modification that has been previously reported 18.

Interestingly, it has been reported that S-acylation of p62 at Cys289/Cys290 potentiates its association with LC3-positive membranes and facilitates the engulfment of p62 condensates, thereby augmenting selective autophagic flux 61. In line with this, inhibition of the deacylating enzyme acyl protein thioesterase 1 ameliorates the HD pathology by restoring brain palmitoylation levels 62. Recent studies have demonstrated that palmitoylation of p62 is essential for autophagy and that this process is impaired in both the brains of HD patients and YAC128 mice, which is reminiscent to p62 UFMylation 14. These observations corroborate our hypothesis that upregulating p62-mediated selective autophagy represents a viable therapeutic strategy for neurodegeneration. Conversely, not all PTMs that enhance the p62-LC3 interaction are beneficial. For instance, elevated nitric oxide levels induce p62 S-nitrosylation at Cys331, which increases the binding of p62 to LC3 but inhibits autophagic flux. This disruption to autophagic flux contributes to the aggregation and dissemination of α-synuclein in Parkinson's disease and Lewy body dementia. Presumably, this impairment stems from the excessive affinity of S-nitrosylated p62 for LC3II, which competitively disrupts the recruitment of fusion machinery to the autophagosomal outer membrane 63.

In this work, we discovered that UFMylation, a previously unrecognized PTM on p62, is critical for the function of p62 in autophagic degradation. However, it remains unclear how p62 UFMylation is dynamically modulated and whether UFMylation intertwines with other modifications under physiological, stressed, or diseased conditions. Further studies are required to answer these questions. Nevertheless, this work paves a new avenue to exploring the diverse biological functions of p62 at the PTM level.

## Materials and Methods

### Reagents and antibodies

Bafilomycin A1 (Baf A1, HY-100558) was purchased from MedChemExpress (USA). Biotin (V900418), chloroquine (CQ, C6628), and DAPI (D9542) were ordered from Sigma-Aldrich (USA). Antibodies were obtained from the following sources: anti-p62 antibody (P0067), anti-polyQ specific monoclonal antibody (MABN2427), and anti-UFL1 antibody (HPA030559) from Sigma-Aldrich; anti-p62 antibody (sc-28359) and anti-ubiquitin antibody (Ub, sc-8017) from Santa Cruz Biotechnology (USA); anti-UFM1 antibody (ab109305) and anti-UFSP2 antibody (ab185965) from Abcam (UK); anti-LC3B antibody (A19665) from ABclonal (China); anti-beclin-1 antibody (20543-1-AP), anti-UBA5 antibody (12093-1-AP), anti-FLAG antibody (20543-1-AP), anti-HA antibody (51064-2-AP), anti-Myc antibody (60003-2-Ig), and anti-GAPDH antibody (60004-1-Ig) from Proteintech Group (China); anti-DARPP32 (2306) antibody from Cell Signaling Technology (USA); Alexa Fluor 488 goat anti-rabbit IgG (AZ1206), Alexa Fluor 488 goat anti-mouse IgG (A11001), Alexa Fluor 594 goat anti-rabbit IgG (A11012), Alexa Fluor 594 goat anti-mouse IgG (A11005), Alexa Fluor 647 goat anti-mouse IgG (A22728), and Alexa Fluor 647 goat anti-rat IgG (A21246) from ThermoFisher Scientific (USA).

### Plasmid construction

Human UBA5, UFC1, UFL1, and UFBP1 were amplified from a cDNA library and cloned into pcDNA3.1-Myc or pcDNA3.1-HA vector. Human and murine p62 were obtained from the corresponding cDNA libraries and cloned into the pHBLV-FLAG, pcDNA3.1-FLAG, and V101-FLAG vectors. UFM1 was cloned into the pHBLV-His_6_-BAP or pcDNA3.1-His_6_ vector. Mutagenesis was performed using a previously described method 64. The sh*p62* sequences were cloned into the pLKO.1-TRC vector (sh*p62* Forward oligo: 5'-CCGGGGCCTACCTTCTGGGCAAGGACTCGAGTCCTTGCCCAGAAGGTAGGCCTTTTTG-3', Reverse oligo: 5'-AATTCAAAAAGGCCTACCTTCTGGGCAAGGACTCGAGTCCTTGCCCAGAAGGTAGGCC-3'). mCherry-GFP-LC3 50 and GFP-Htt60Q plasmids 53 were kindly provided by Dr. Guanghui Wang from Soochow University.

### siRNA and plasmid transfection

siRNA sequences used in this work were: si*UBA5*#1: 5'-CGUACCUUUGCCGUAGCAA-3'; si*UBA5*#2: 5'-CUGAAACGAGAAGGTGUUU-3'. si*UFL1*#1: 5'-GGAACUUGUUAAUAGCGGA-3'; si*UFL1*#2: 5'-GAGGAGUAAUUUUUACGGA-3'. HeLa and MCF-7 cells were transfected with si*NC* (160818) or target siRNA using riboFECT^TM^ CP transfection Kit (C1051105, RiboBio, China) according to a previous publication 65. Lipofectamine™ 2000 transfection reagent (11668019, Invitrogen, USA) was used to transfect plasmids into cells.

### Cell culture

HEK293T, HeLa, and MCF-7 cells were obtained from American Type Culture Collection (ATCC, USA). HEK293T-sg*UFSP2* and HEK293T-sg*Ctrl* cells were kindly provided by Dr. Yu-sheng Cong from Hangzhou Normal University 28. Cells were cultured in DMEM or RPMI 1640 supplemented with 10% FBS, penicillin, and streptomycin, and placed in a cell culture incubator with 5% CO_2_ and full humidity at 37 °C.

### Cortical neuron culture

The C57BL/6 mouse brains from embryos at E16 were aseptically collected. The cerebral cortices were isolated under a microscope and digested with 0.25% trypsin, which was stopped by adding Neurobasal medium (21103049, Gibco) supplemented with DNase I (5 mg/mL, DN25, Sigma-Aldrich). Cells were separated and cultured in Neurobasal medium containing 2% B-27 (17504044, Gibco), 2 mM L-glutamine (25030164, Gibco), and 50 U/mL penicillin and streptomycin. Then, the primary cortical neurons were cultured in poly-L-lysine-coated plates (0.1 mg/mL) for one week before subsequent experiments.

### Lentiviral infection

HeLa cells were treated with lentiviral particles and supplemented with 10 μg/mL polybrene for 12 h. The *p62* knockdown stable cell lines were selected with puromycin for two weeks, and the knockdown efficiency of target genes was examined by immunoblotting. The primary cortical neurons were infected with lentivirus for 48 h for subsequent experiments.

### Quantitative PCR (qPCR)

qPCR was performed according to a published method 66. Primers with the following sequences were synthesized by GENEWIZ Biotech (China). *LC3B* Forward: 5'-GGATATAGGTCACCCCTCAG-3', Reverse: 5'-GTTAAAGGAGTTCCTGTCACC-3'; *GAPDH* Forward: 5'-TGCACCACCAACTGCTTAGC-3', Reverse: 5'-ACAGTCTTCTGGGTGGCAGTG-3'. *GAPDH* was used for normalization.

### Affinity purification

Anti-FLAG affinity gel (B23102, Bimake, USA) was used to purify FLAG-tagged proteins following a previous approach 67. Cell lysate was incubated with prewashed anti-FLAG affinity gel at 4 °C overnight. The gel was washed thrice with RIPA buffer. Proteins were eluted twice with 200 µg/mL FLAG peptide and combined for subsequent analysis.

Ni-NTA resin (30410, QIAGEN, Germany) and NeutrAvidin agarose resin (29201, ThermoFisher Scientific, USA) were used sequentially to purify the UFMylated proteins based on the previously published procedure 68. Cell lysates were incubated with Ni-NTA resin under denaturing conditions. Purified proteins were eluted with 300 mM imidazole. Ni-NTA eluate was subsequently incubated with NeutrAvidin agarose resin at 4 °C overnight followed by washing. Proteins were eluted as described previously 64.

### Protein sample preparation and MS analysis

Protein samples for MS analyses were processed as previously described 68. Proteins were resolved by SDS-PAGE and gel bands were excised into small pieces. Proteins were trypsinized in-gel after reduction and alkylation. Peptide samples were subjected to an Orbitrap Fusion Lumos mass spectrometer equipped with an electrospray ionization inlet (ThermoFisher Scientific) as described previously 69.

LC-MS/MS raw files were analyzed with Proteome Discoverer (ThermoFisher Scientific) against the human protein database, containing decoy protein sequences and common contaminants. The methionine oxidation, N-terminal acetylation, and UFM1 remnant (Val-Gly, obtained after trypsin cleavage of the peptide bond after Arg near the UFM1 C-terminus) on Lys were set as variable modifications. Log_2_ (Fold Change) (UFM1/pHBLV) was obtained based on the protein abundance in the pHBLV-His_6_-BAP-UFM1 and pHBLV expressing samples. *P*-value was calculated with a two-tailed Student's *t*-test for data from three biological replicates. Volcano plot was constructed in Origin 9 using Log_2_ (Fold Change) and -Log_10_ (*P*-value).

### Immunoblotting

Immunoblotting was conducted following a published method 70, 71. In brief, proteins were separated by SDS-PAGE and transferred to a piece of PVDF membrane, which was blocked, and incubated with primary and secondary antibodies. Chemiluminescent signals were imaged and analyzed using a Tanon 5200 imaging system (China).

### Mouse brain tissue preparation and dissection

Twelve weeks after injection of the adeno-associated virus, mice were perfused with PBS and 4% paraformaldehyde, and then brains were cryoprotected in 30% sucrose solution. Serial brain sections with a thickness of 20 μm were prepared using a freezing microtome (Leica, CM1950, Germany).

### Immunofluorescence

Immunofluorescence was performed according to established protocols 66. Cells were fixed, permeabilized, blocked, incubated with primary antibodies overnight at 4 °C, and stained with Alexa Fluor 488 goat anti-rabbit IgG, Alexa Fluor 594 goat anti-rabbit IgG, or Alexa Fluor 647 goat anti-mouse IgG for 2 h in the dark. Primary cortical neurons were incubated with primary antibody overnight at 4 °C, and then with Alexa Fluor 594 goat anti-rabbit IgG, Alexa Fluor 488 goat anti-mouse IgG, and Alexa Fluor 647 goat anti-rat IgG for 2 h in the dark. The embedded mouse brain was dissected into 20 μm sections using a freezing microtome, and the slices were blocked and probed with primary antibodies overnight at 4 °C, and then incubated with Alexa Fluor 594 goat anti-rabbit IgG or Alexa Fluor 594 goat anti-mouse IgG for 2 h in the dark. Nuclei were stained with DAPI for 10 min. Images were taken with the Nikon A1R HD25 confocal microscope (Japan).

### *In vitro* UFMylation assay

Purified UBA5 (2 μM), UFC1 (2 μM), UFL1 (2 μM), UFM1^ΔC2^ (2 μM), and GST-p62 (2 μM) or GST-p62^2KR^ (2 μM) were mixed in the reaction buffer (5 mM ATP, 0.05% BSA, 10 mM MgCl_2_, 50 mM HEPES, pH 7.5) and incubated at 30 ℃ for 2 h. The sample was mixed with 5 × SDS sample loading buffer and heated at 98 ºC for 10 min.

### Autophagic flux assay

Autophagy flux assay was performed using a published procedure 50. Cells transfected with mCherry-GFP-LC3 were fixed, blocked, stained with DAPI, and washed thrice with PBS. Microphotographs of mCherry-GFP-LC3 fluorescence were captured under a confocal microscope. Autophagic flux was evaluated with ImageJ by counting the number of yellow and red puncta for 30 cells. Specifically, the Analyze Particles function in ImageJ was activated, the particle size threshold was set to 300-infinity pixels^2^, and the Exclude on Edges option was enabled.

### Sample preparation for transmission electron microscopy

Cells were fixed with glutaraldehyde (2.5%) overnight at 4 °C as described previously 72, stained with osmium tetroxide (0.1 M) in PBS for 2 h, and rinsed thrice with PBS. Then, cells were dehydrated with a gradient concentration of ethanol (30%, 50%, 70%, 80%, 85%, 90%, 95%, 100%) for 15 min each and infiltrated with epoxy resin for 12 h at 37 °C. After epoxy resin was embedded and polymerized for 48 h at 60 ºC, 80-nm-thick ultrathin sections were prepared using a diamond knife on a Leica EM UC7 ultramicrotome, and double-stained with 2% aqueous uranyl acetate and aqueous lead citrate for 15 min. Images were taken with a transmission electron microscope (Hitachi HT7700, Japan).

### Animals and stereotaxic viral injection

FVB-Tg (YAC128)/Nju mice and WT littermates were purchased from Model Animal Research Center, MARC (China). B6-hHTT130-N mice (Strain #: T054804) were purchased from GemPharmatech Co., Ltd (Nanjing, China). YAC128 is a mouse model expressing the full-length human *HTT* gene with 128 CAG repeats. The B6-hHTT130-N mouse model incorporates a human HTT gene fragment carrying a 130 CAG repeat expansion with the mouse *Htt* promoter. Mice were fed with a standard chow diet and free access to water, and maintained in a standard SPF facility. Adeno-associated viruses of rAAV-hSyn-EGFP, rAAV-hSyn-mp62-P2A-EGFP, rAAV-hSyn-mp62^2KR^-P2A-EGFP, and rAAV-hSyn-mp62^2KR^-mUFM1^ΔC4^-P2A-EGFP were purchased from BrainCase (China). Eight-week-old B6-HTT-CAG130-N mice were placed on a stereotaxic instrument after being anesthetized. Adeno-associated virus (2 μL) was injected into the striatum using a 5 μL Hamilton microsyringe with an injection rate of 0.5 μL/min (anteroposterior = -1.0 mm, mediolateral = ±2.0 mm, dorsoventral = -3.3 mm from bregma). After the completion of the injection, the needle was maintained in the same position for 5 min and postsurgical care was provided for the mice. After the experiment, mice were euthanized and tissues were harvested. The animal procedure was approved by the Ethics Committee of Soochow University (SUDA20240911A28).

### Statistical analysis

Data from Western blotting and qPCR were analyzed using unpaired Student's *t*-test or one-way ANOVA with Tukey's post hoc test for multiple comparisons. For the autophagic flux assay, statistics between multiple groups were performed using two-way ANOVA with Tukey's post hoc test in GraphPad Prism Software (San Diego, CA).

## Supplementary Material

Supplementary figures.

Supplementary table 1.

Supplementary table 2.

Supplementary table 3.

## Abbreviations

ALP: autophagy-lysosome pathway
Baf A1: bafilomycin A1
CQ: chloroquine
HTT: huntingtin
HD: Huntington's disease
LC3: microtubule-associated protein 1A/1B-light chain 3
LIR: LC3-interacting region
mHTT: mutant Huntingtin
MS: mass spectrometry
MSN: medium spiny neuron
PTM: post-translational modification
UBA: ubiquitin-association
UBA5: UFM1-activating enzyme 5
UFBP1: UFM1-binding and PCI domain-containing protein 1 or DDRGK domain-containing protein 1 (DDRGK1)
UFC1: UFM1-conjugating enzyme 1
UFL1: UFM1-protein ligase 1
UFSP1/2: UFM1 specific proteases 1/2
WT: wild-type
