# Supplementary material for: SQSTM1/p62 UFMylation Enhances Autophagic Clearance of Pathogenic Mutant Huntingtin
Source: Int J Biol Sci. 2026 May 18;22(10):5475–94. doi: 10.7150/ijbs.127110 (PMC13215360; doi:10.7150/ijbs.127110)
Supplement: Supplementary file 1 — Supplementary figures. [file ijbsv22p5475s1.pdf]

Supplementary Figures for

SQSTM1/p62 UFMylation Enhances Autophagic Clearance of Pathogenic Mutant Huntingtin

Xiaohui Wang, Xiaowei Lv, Honglv Jiang, Wenyun Zhu, Lindong Cao, Liang Zhou, Fang Lin, Rong Deng, Li-Fang Hu, Jingjing Ma*, Jia-Bin Li*, and Guoqiang Xu*

This file includes:

Figure S1 to S9.

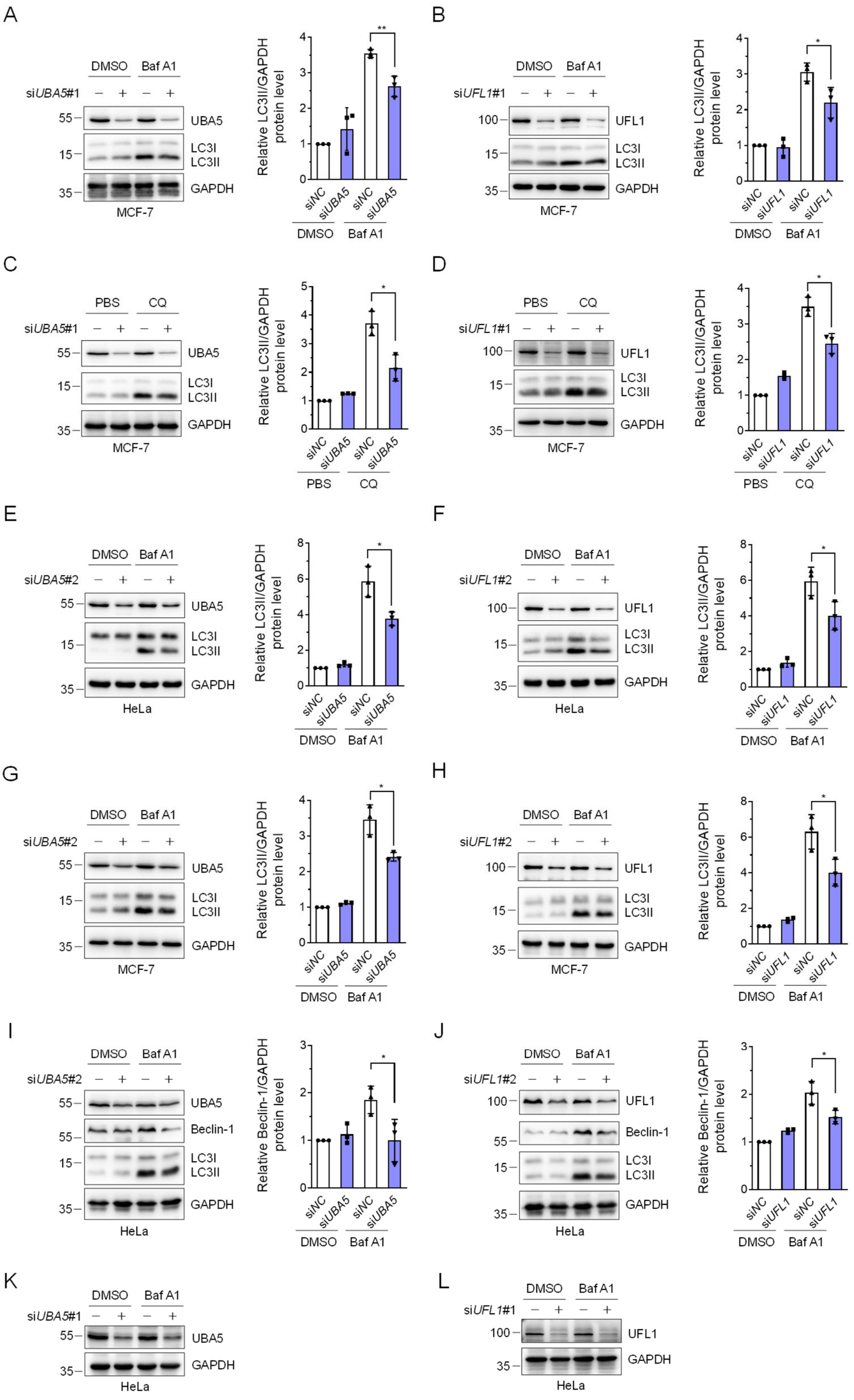

Figure S1. Knockdown of *UFL1* or *UBA5* blocks autophagic activity. (**A-D**) Control (si*NC*) and *UBA5*- or *UFL1*-knockdown (si*UBA5*#1 or si*UFL1*#1) MCF-7 cells were

treated with Baf A1 (200 nM for 4 h) (A-B) or CQ (50 μM for 12 h) (C-D). Cell lysates were immunoblotted for the indicated proteins. Mean ± SD, n = 3 biological replicates, two-way ANOVA with Tukey's multiple comparisons post hoc test. *: $P < 0.05$, **: $P < 0.01$. (**E-H**) Control (si*NC*) and *UBA5*- (E, G) or *UFL1*- (F, H) knockdown (si*UBA5*#2 or si*UFL1*#2) HeLa or MCF-7 cells were treated with Baf A1 (200 nM for 6 h) and cell lysates were immunoblotted for LC3, UBA5, or UFL1. The relative protein levels of LC3II were normalized to GAPDH and quantified. Mean ± SD, n = 3 biological replicates, two-way ANOVA with Tukey's multiple comparisons post hoc test. *: $P < 0.05$. (**I-J**) Control (si*NC*) and *UBA5*- (I) or *UFL1*- (J) knockdown (si*UBA5*#2 or si*UFL1*#2) HeLa cells were treated with Baf A1 (200 nM) for 6 h and cell lysates were immunoblotted for the indicated proteins. The relative protein levels of LC3II were normalized to GAPDH and quantified. Mean ± SD, n = 3 biological replicates, two-way ANOVA with Tukey's multiple comparisons post hoc test, *: $P < 0.05$. (**K-L**) Immunoblotting of cell lysates obtained from Figure 1E and 1F. GAPDH was used as the loading control.

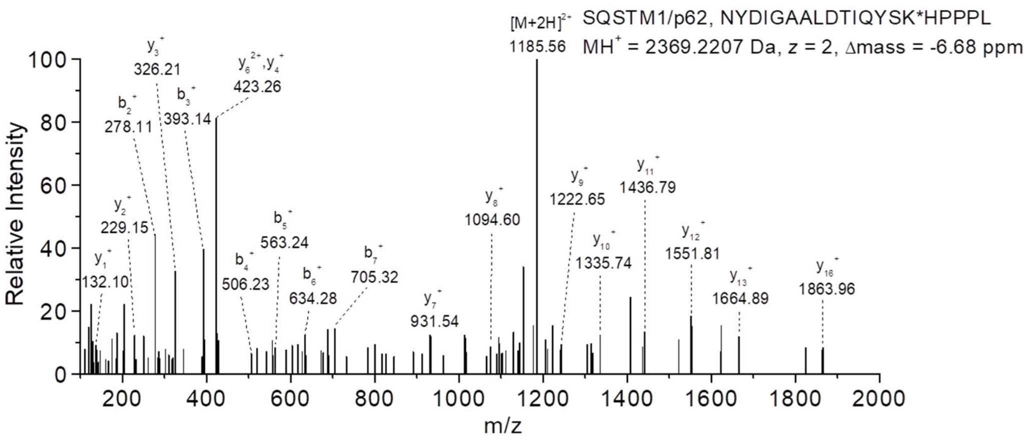

Figure S2. MS/MS spectrum of the p62 tryptic peptide containing the UFMylation site. The peptide sequence, MH^+, charge state (z), and Δmass (ppm) were provided. K*: VG modified lysine.

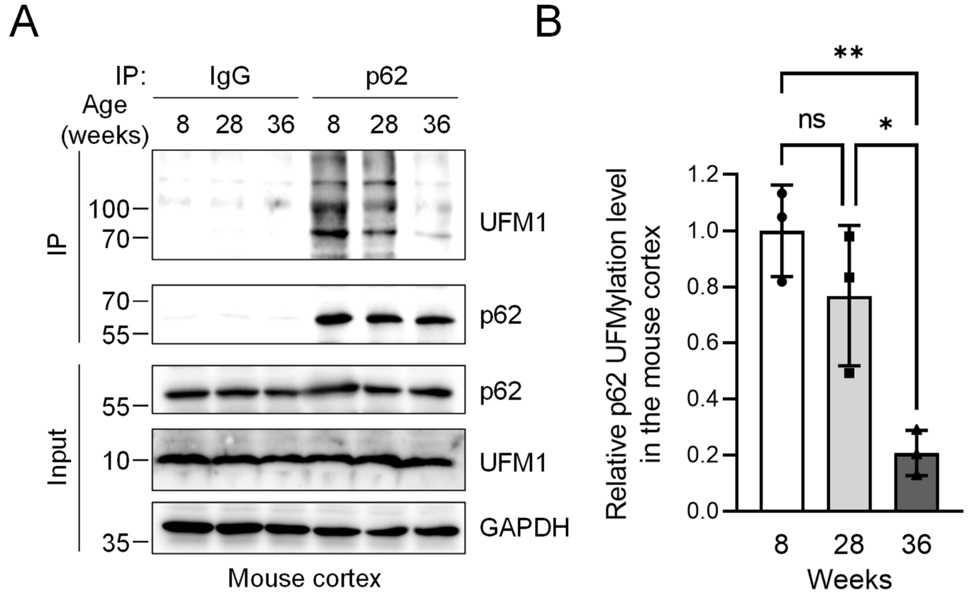

Figure S3. Endogenous p62 in the mouse cortex is UFMylated. (**A-B**) Cortexes from 8, 28, or 36-week-old mice were dissected and lysed. The endogenous p62 was immunoprecipitated with IgG or anti-p62 antibodies. Lysates and immunoprecipitates were immunoblotted with the indicated antibodies (A) and the relative p62 UFMylation level was quantified (B). Mean ± SD, n = 3 biological replicates, one-way ANOVA with Tukey's multiple comparisons post hoc test, *: $P < 0.05$, **: $P < 0.01$, ns: not significant.

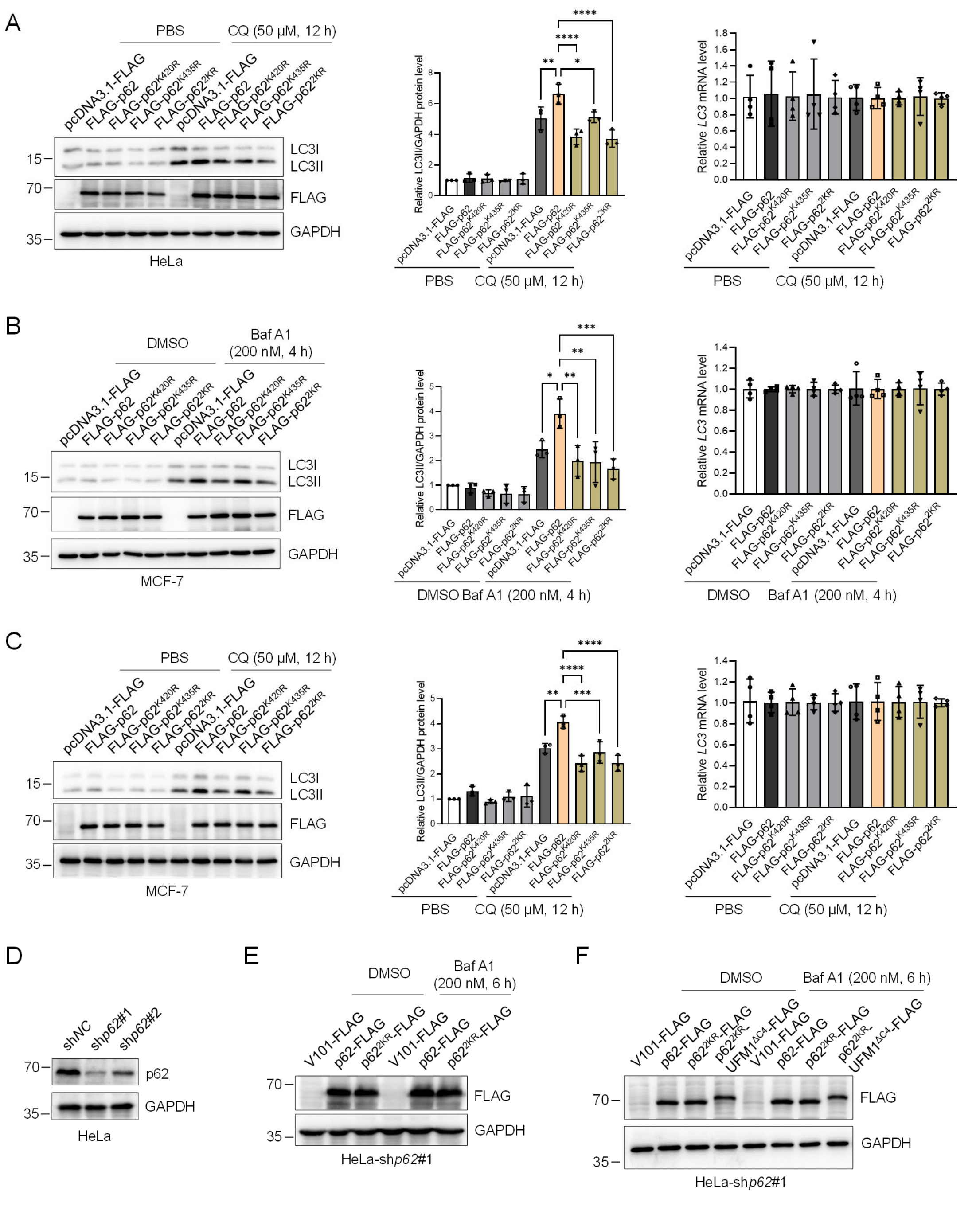

Figure S4. UFMylation-deficient p62 mutant abolishes the effect of p62 on LC3. (A-C) HeLa or MCF-7 cells were transfected with pcDNA3.1-FLAG, FLAG-p62, FLAG-p62^{K420R}, FLAG-p62^{K435R} or FLAG-p62^{2KR} plasmid for 48 h and then treated with CQ (50 μM for 12 h) (A), Baf A1 (200 nM for 4 h) (B), and CQ (50 μM for 12 h) (C). Left

panels: Immunoblotting analysis of cell lysates. Middle panels: Quantification of immunoblotting results from three biological replicates. Right panels: qPCR analysis of *LC3* mRNA. The relative protein levels of LC3II were normalized to GAPDH and quantified. Immunoblotting data were presented as mean ± SD (n = 3 biological replicates) and qPCR data were obtained from four technical replicates. Similar results were obtained from two biological replicates of qPCR experiments. Two-way ANOVA with Tukey's multiple comparisons post hoc test, *: $P < 0.05$, **: $P < 0.01$, ***: $P < 0.001$, ****: $P < 0.0001$. (**D**) Verification of *p62* knockdown efficiency in HeLa cells. (**E-F**) Cell lysates from Figure 4I and 5J were subjected to immunoblotting analysis.

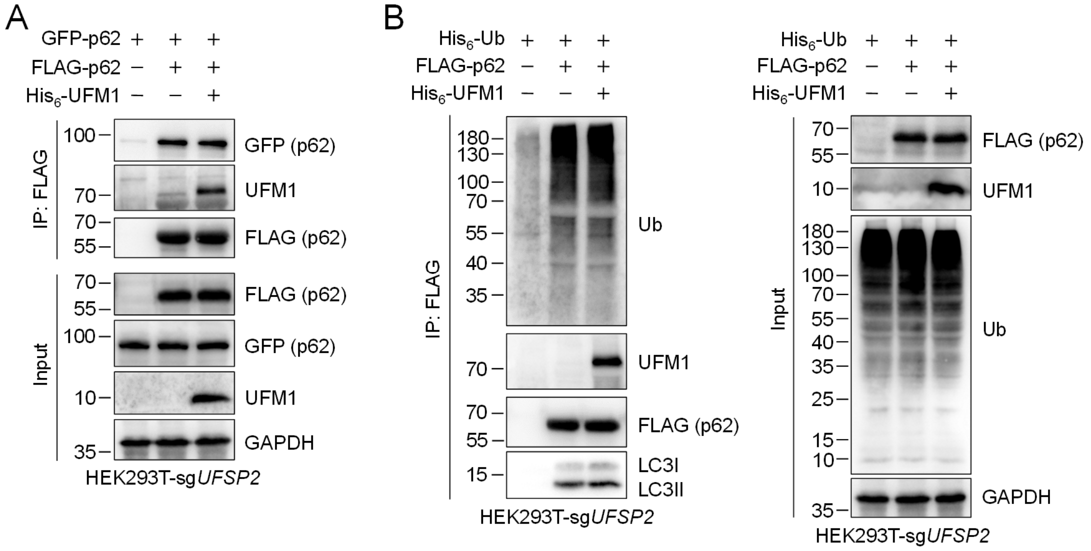

Figure S5. UFM1 does not affect the p62 oligomerization and the interaction between p62 and ubiquitinated proteins. (**A**) UFM1 does not affect p62 oligomerization. HEK293T-sg*UFSP2* cells were transfected with GFP-p62, FLAG-p62, and/or His_6-UFM1 plasmids for 48 h. p62 was purified with anti-FLAG affinity gel and eluted with FLAG peptide (200 μg/mL). The cell lysates and affinity-purified samples were immunoblotted. (**B**) UFM1 does not influence the association of p62 with ubiquitinated proteins. HEK293T-sg*UFSP2* cells were transfected with His_6-Ub, FLAG-p62, and His_6-UFM1 plasmids for 36 h, and treated with MG132 (10 μM) for 12 h. Then, p62 was purified with anti-FLAG affinity gel and eluted with FLAG peptide (200 μg/mL). The cell lysates and affinity-purified samples were immunoblotted.

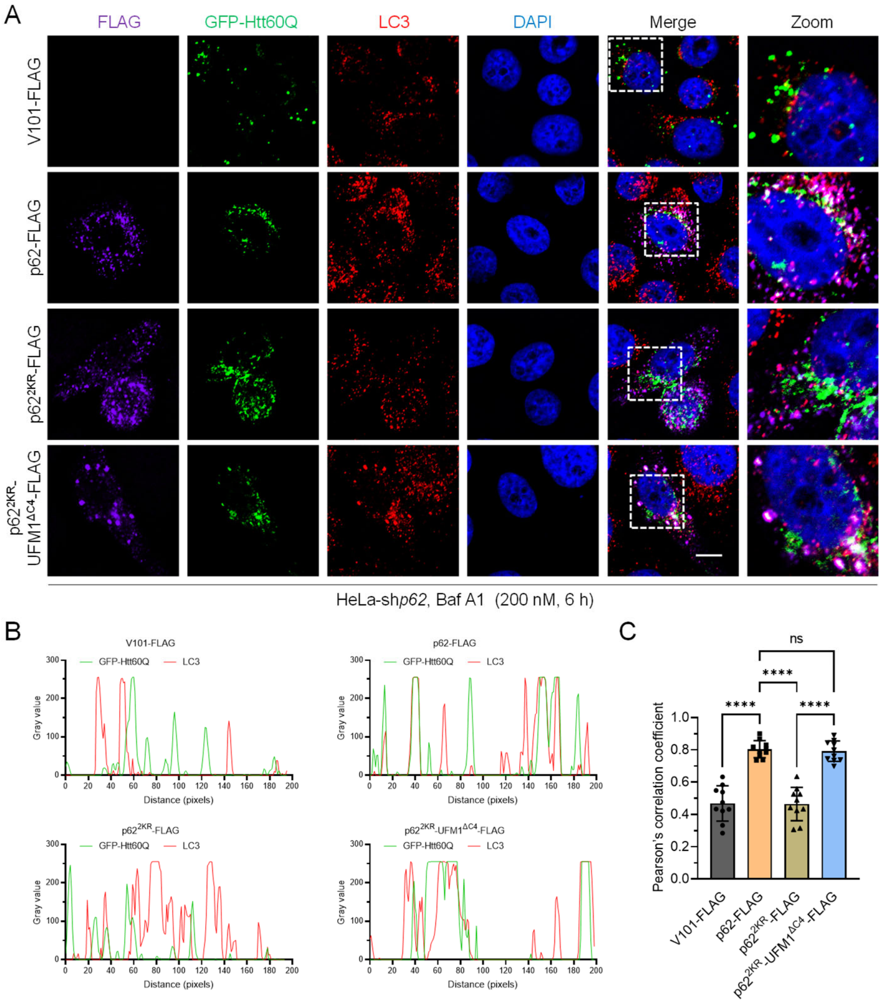

Figure S6. The UFMylation of p62 promotes the colocalization of mHtt60Q with LC3 puncta. (**A-B**) The *p62*-knockdown HeLa cells were transfected with V101-FLAG, p62-FLAG, p62^{2KR}-FLAG, or p62^{2KR}-UFM1$^{\Delta C4}$-FLAG, and GFP-Htt60Q plasmids for 42 h, treated with Baf A1 (200 nM) for 6 h, and then stained with FLAG, LC3, and DAPI. Representative confocal images (A) and colocalization analysis of GFP-Htt60Q with LC3 (B). Scale bar, 10 μm. (**C**) The Pearson's correlation coefficient for the colocalization of GFP-Htt60Q and LC3. Mean ± SD (n = 3 biological replicates with 10 cells), one-way ANOVA with Tukey's multiple comparisons post hoc test, ****: $P < 0.0001$, ns: not significant.

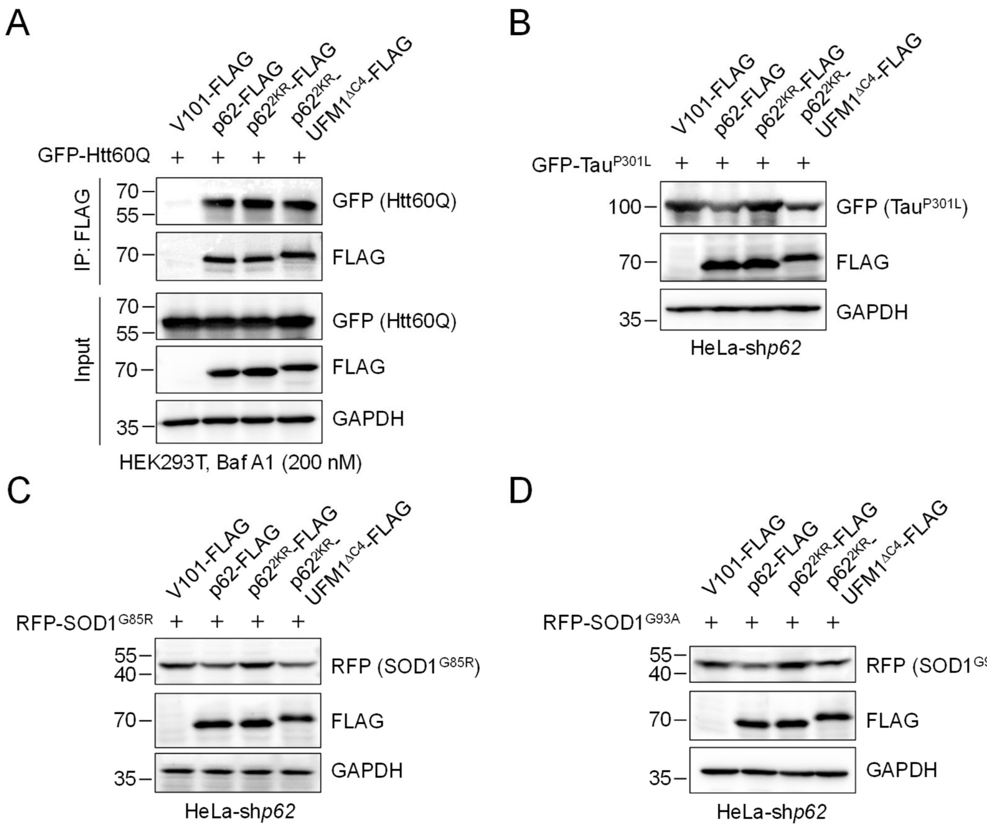

Figure S7. The UFMylation of p62 is not required for substrate interaction but essential for the degradation of neurodegeneration-associated proteins. (**A**) The UFMylation of p62 does not affect its binding to mHTT. HEK293T cells were transfected with V101-FLAG, p62-FLAG, p62^{2KR}-FLAG, or p62^{2KR}-UFM1$^{\Delta C4}$-FLAG, and GFP-Htt60Q plasmids for 42 h, treated with Baf A1 (200 nM) for 6 h, and then p62 was purified with anti-FLAG affinity gel and eluted with FLAG peptide (200 μg/mL). The cell lysates and affinity-purified samples were immunoblotted. (**B-D**) The UFMylation of p62 promotes the degradation of neurodegeneration-associated proteins. The *p62*-knockdown HeLa cells were transfected with V101-FLAG, p62-FLAG, p62^{2KR}-FLAG, or p62^{2KR}-UFM1$^{\Delta C4}$-FLAG, and GFP-Tau^{P301L} (**B**), RFP-SOD^{G85R} (**C**), or RFP-SOD1^{G93A} (**D**) plasmids for 48 h. Then the cell lysates were subjected to immunoblotting analysis.

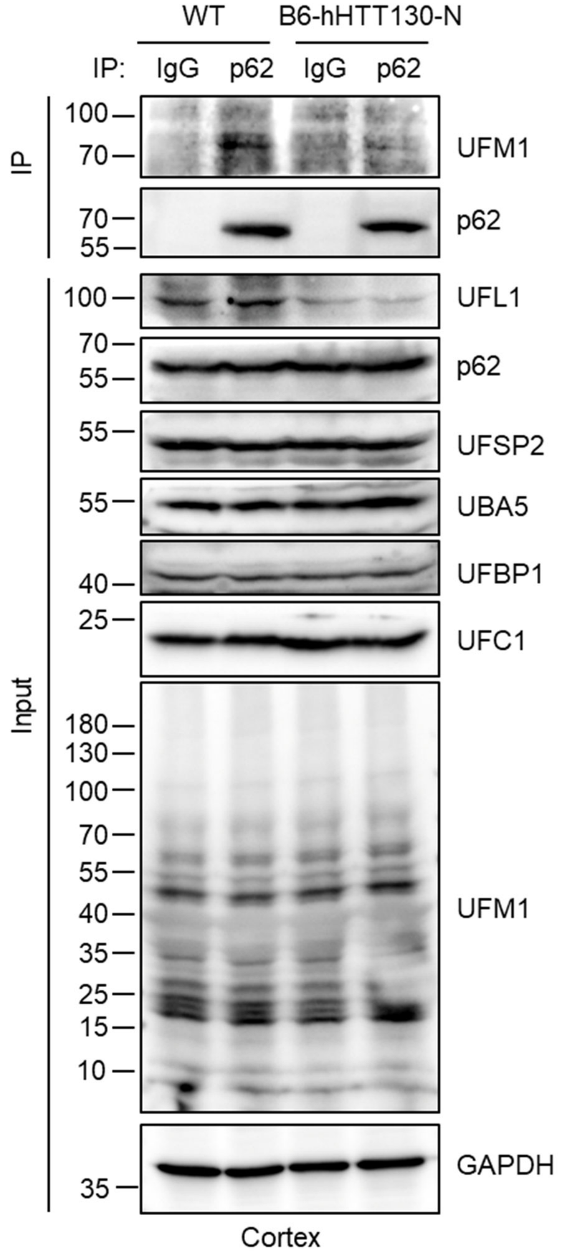

Figure S8. p62 UFMylation is decreased in the cortex of B6-hHTT130-N. The cortex of WT or B6-hHTT130-N mice was lysed and endogenous p62 was immunoprecipitated with IgG or anti-p62 antibodies for immunoblotting analysis.

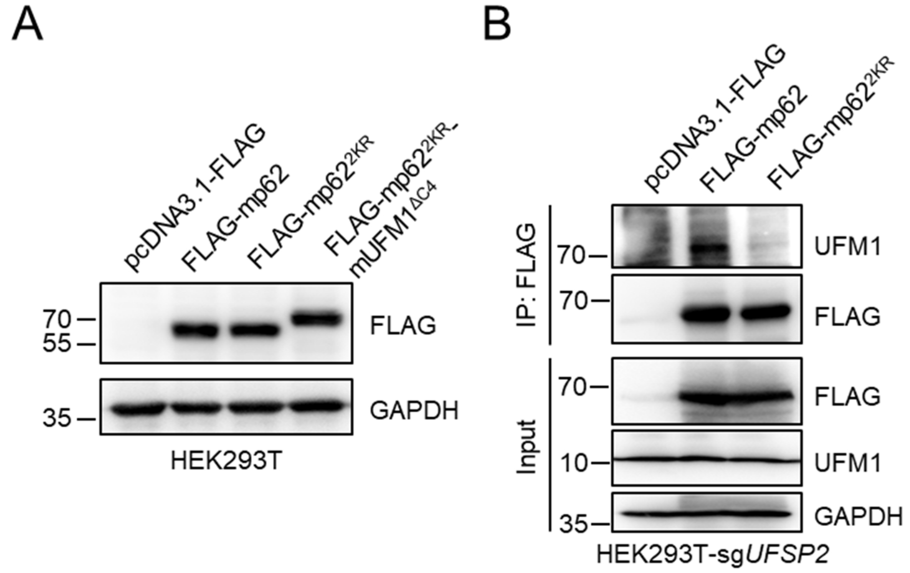

Figure S9. Expression and UFMylation of murine p62 and its mutants. (**A**) The expression of murine wild-type FLAG-mp62, UFMylation-deficient mutant FLAG-mp62^{2KR} (K422R and K437R), and FLAG-mp62^{2KR}-mUFM1$^{\Delta C4}$ in HEK293T cells was determined by immunoblotting. (**B**) Biochemical validation of murine p62 UFMylation and its UFMylation sites. HEK293T-sg*UFSP2* cells were transfected with His_6-UFM1 and pcDNA3.1-FLAG, FLAG-mp62, or FLAG-mp62^{2KR} (K422R and K437R) for 48 h. mp62 was purified with anti-FLAG affinity gel and eluted with FLAG peptide (200 μg/mL). The cell lysates and purified samples were immunoblotted.
